# Supplementary material for: Test accuracy of faecal calprotectin for inflammatory bowel disease in UK primary care: a retrospective cohort study of the THIN data
Source: BMJ Open. 2021 Feb 22;11(2):e044177. doi: 10.1136/bmjopen-2020-044177 (PMC7903095; doi:10.1136/bmjopen-2020-044177)
Supplement: Supplementary data [file bmjopen-2020-044177supp001.pdf]

SUPPLEMENT

Supplementary Table 1 Final variable code lists

Variable	medcode	description
IBD	14C4.11	H/O: ulcerative colitis
	J08z900	Orofacial Crohn's disease
	J4...12	Inflammatory bowel disease
	J40..00	Regional enteritis - Crohn's disease
	J40..11	Crohn's disease
	J40..12	Granulomatous enteritis
	J400.00	Regional enteritis of the small bowel
	J400000	Regional enteritis of the duodenum
	J400100	Regional enteritis of the jejunum
	J400200	Crohn's disease of the terminal ileum
	J400300	Crohn's disease of the ileum unspecified
	J400400	Crohn's disease of the ileum NOS
	J400500	Exacerbation of Crohn's disease of small intestine
	J400z00	Crohn's disease of the small bowel NOS
	J401.00	Regional enteritis of the large bowel
	J401000	Regional enteritis of the colon
	J401100	Regional enteritis of the rectum
	J401200	Exacerbation of Crohn's disease of large intestine
	J401z00	Crohn's disease of the large bowel NOS
	J401z11	Crohn's colitis
	J402.00	Regional ileocolitis
	J40z.11	Crohn's disease NOS
	J41..12	Ulcerative colitis and/or proctitis
	J410.00	Ulcerative proctocolitis
	J410000	Ulcerative ileocolitis
	J410100	Ulcerative colitis
	J410200	Ulcerative rectosigmoiditis
	J410300	Ulcerative proctitis
	J410400	Exacerbation of ulcerative colitis
	J410z00	Ulcerative proctocolitis NOS
	J411.00	Ulcerative (chronic) enterocolitis
	J412.00	Ulcerative (chronic) ileocolitis
	J413.00	Ulcerative pancolitis
	J436.00	Microscopic colitis
	J436000	Collagenous colitis
	J436100	Lymphocytic colitis
	J438.00	Left sided colitis
	J4z3.00	Non-infective colitis NOS
	J4z4.00	Non-infective sigmoiditis NOS
	J4z6.00	Indeterminate colitis

	Jyu4000	[X]Other Crohn's disease
	Jyu4100	[X]Other ulcerative colitis
	N031000	Arthropathy in ulcerative colitis
	N031100	Arthropathy in Crohn's disease
	N045300	Juvenile arthritis in Crohn's disease
	N045400	Juvenile arthritis in ulcerative colitis
	ZR3S.00	Crohn's disease activity index
	ZR3S.11	CDAI - Crohn's disease activity index
IBS	14CF.00	History of irritable bowel syndrome
	8CA4a00	Education about FODMAP exclusion diet
	8CA4Z00	Dietary education for irritable bowel syndrome
	8Cm..00	Management of irritable bowel syndrome
	Eu45324	[X]Psychogenic IBS
	J521.00	Irritable colon - Irritable bowel syndrome
	J521.11	Irritable bowel syndrome
	J521.13	Spastic colon
	J521000	Irritable bowel syndrome with diarrhoea
	J521100	Irritable bowel syndrome characterised by constipation
	J521200	IBS characterised by alternating bowel habit
Colorectal cancer	68W2400	Bowel scope (flexible sigmoidoscopy) screen: cancer detected
	68W2500	Bowel scope (flexi-sig) screen: suspected cancer detected
	9Ow1.00	Bowel cancer detected by national screening programme
	B13..00	Malignant neoplasm of colon
	B130.00	Malignant neoplasm of hepatic flexure of colon
	B131.00	Malignant neoplasm of transverse colon
	B132.00	Malignant neoplasm of descending colon
	B133.00	Malignant neoplasm of sigmoid colon
	B134.00	Malignant neoplasm of caecum
	B134.11	Carcinoma of caecum
	B136.00	Malignant neoplasm of ascending colon
	B137.00	Malignant neoplasm of splenic flexure of colon
	B138.00	Malignant neoplasm, overlapping lesion of colon
	B139.00	Hereditary nonpolyposis colon cancer
	B13y.00	Malignant neoplasm of other specified sites of colon
	B13z.00	Malignant neoplasm of colon NOS
	B13z.11	Colonic cancer
	B14..00	Malignant neoplasm of rectum, rectosigmoid junction and anus
	B140.00	Malignant neoplasm of rectosigmoid junction
	B141.00	Malignant neoplasm of rectum
	B141.11	Carcinoma of rectum
	B141.12	Rectal carcinoma
	B142000	Malignant neoplasm of cloacogenic zone

	B14y.00	Malig neop other site rectum, rectosigmoid junction and anus
	B14z.00	Malignant neoplasm rectum,rectosigmoid junction and anus NOS
	B1z0.11	Cancer of bowel
	B575.00	Secondary malignant neoplasm of large intestine and rectum
	B575000	Secondary malignant neoplasm of colon
	B575100	Secondary malignant neoplasm of rectum
	B575z00	Secondary malig neop of large intestine or rectum NOS
	B902400	Neoplasm of uncertain behaviour of colon
	B902500	Neoplasm of uncertain behaviour of rectum
	BB5N.00	[M]Adenomatous and adenocarcinomatous polyps of colon
	BB5N.11	[M]Adenoma or or adenocarcinoma in polyposis coli
	BB5N100	[M]Adenocarcinoma in adenomatous polposis coli
	BB5Nz00	[M]Adenomatous or adenocarcinomatous polyps of the colon NOS
	ZV10017	[V]Personal history of malignant neoplasm of rectum
Change in bowel habit	19EA.00	Change in bowel habit
	19EA.11	Altered bowel habit
	19EE.00	Increased frequency of defaecation
	J521200	IBS characterised by alternating bowel habit
	R078.00	[D]Change in bowel habit
Constipation	19C..00	Constipation
	19C..11	Constipation symptom
	19C..12	Costive symptom
	19C2.00	Constipated
	19CZ.00	Constipation NOS
	E264500	Psychogenic constipation
	J520.00	Constipation - functional
	J520000	Acute constipation
	J520100	Chronic constipation with overflow
	J520200	Chronic constipation without overflow
	J520400	Chronic constipation
	J520y00	Other specified constipation
	J520z00	Constipation NOS
	J521100	Irritable bowel syndrome characterised by constipation
	J52y100	Difficulty in ability defaecat
Diarrhoea	19E3.00	Incontinent of faeces
	19E3.11	Incontinent of faeces symptom
	19EE.00	Increased frequency of defaecation
	19EF.00	Urgent desire for stool
	19F..00	Diarrhoea symptoms
	19F..11	Diarrhoea

	19F..12	Loose stools
	19F2.00	Diarrhoea
	19F3.00	Spurious (overflow) diarrhoea
	19F5.00	Time since last episode of diarrhoea
	19FZ.00	Diarrhoea symptom NOS
	19FZ.11	Diarrhoea & vomiting, symptom
	19G..00	Diarrhoea and vomiting
	E264300	Psychogenic diarrhoea
	E264311	Spurious diarrhoea
	Eu45317	[X]Psychogenic diarrhoea
	J4...13	Noninfective diarrhoea
	J43z.11	Chronic diarrhoea
	J4z..11	Presumed noninfectious diarrhoea
	J4zz.11	Diarrhoea - presumed non-infectious
	J521000	Irritable bowel syndrome with diarrhoea
	J525.00	Functional diarrhoea
	J528.00	Intestinal hurry
	R077100	[D] Stools loose
Bloating	19A..00	Abdominal distension symptom
	19A2.00	Abdomen feels bloated
	19A3.00	Abdomen feels distended
	19A4.00	Abdomen feels swollen
	19AZ.00	Abd. distension symptom NOS
	19B..00	Flatulence/wind
	19B..12	Bloating symptom
	19B..14	Flatulence symptom
	19B..15	Wind symptom
	19B2.00	Excessive flatulence
	19B5.00	Excessive flatus
	19BZ.00	Wind NOS
	Eu45320	[X]Psychogenic flatulence
	R073.00	[D]Flatulence, eructation and gas pain
	R073000	[D]Flatulence
	R073200	[D]Gas pain (abdominal)
	R073300	[D]Abdominal distension, gaseous
	R073400	[D]Bloating
	R073500	[D]Tympanites (abdominal)
	R073z00	[D]Flatulence, eructation and gas pain NOS
	R073z11	[D]Wind
Abdominal pain	196..11	Abdominal pain type
	1962.00	Colicky abdominal pain
	1963.00	Non-colicky abdominal pain

	1967.00	Abdominal migraine - symptom
	1968.00	Abdominal discomfort
	1969.00	Abdominal pain
	1969000	Abdominal wall pain
	197..11	Flank pain
	197..12	Iliac fossa pain
	197..13	Site of abdominal pain
	197..14	Subcostal pain
	1971.00	Central abdominal pain
	1972.00	Epigastric pain
	1973.00	Left subcostal pain
	1974.00	Right subcostal pain
	1975.00	Left flank pain
	1976.00	Right flank pain
	1977.00	Right iliac fossa pain
	1978.00	Left iliac fossa pain
	1979.00	Suprapubic pain
	197A.00	Generalised abdominal pain
	197A.11	General abdominal pain-symptom
	197B.00	Upper abdominal pain
	197C.00	Lower abdominal pain
	197D.00	Right upper quadrant pain
	25C..00	O/E - abdo. pain on palpation
	25C..11	O/E - epigastric pain on palp.
	25C..12	O/E - iliac pain on palpation
	25C..14	O/E - umbilical pain on palp.
	25C..15	O/E - abdomen tender
	25C2.00	O/E - abd.pain-R.hypochondrium
	25C3.00	O/E - abd. pain - epigastrium
	25C4.00	O/E - abd.pain-L.hypochondrium
	25C5.00	O/E - abd. pain - R.lumbar
	25C6.00	O/E - abd. pain - umbilical
	25C7.00	O/E - abd. pain - L.lumbar
	25C8.00	O/E - abd. pain - R.iliac
	25C9.00	O/E - abd. pain - hypogastrium
	25CA.00	O/E - abd. pain - L.iliac
	25CZ.00	O/E -abd.pain on palpation NOS
	F262200	Abdominal migraine
	R073200	[D]Gas pain (abdominal)
	R090.00	[D]Abdominal pain
	R090000	[D]Abdominal tenderness
	R090100	[D]Abdominal colic
	R090400	[D]Abdominal cramps
	R090500	[D]Epigastric pain

	R090600	[D]Umbilical pain
	R090700	[D]Hypochondrial pain
	R090800	[D]Suprapubic pain
	R090900	[D]Pain in right iliac fossa
	R090A00	[D]Pain in left iliac fossa
	R090C00	[D]Loin pain
	R090D00	[D]Abdominal migraine
	R090E00	[D]Recurrent acute abdominal pain
	R090F00	[D]Acute abdomen
	R090G11	[D] Pelvic pain
	R090H00	[D]Upper abdominal pain
	R090J00	[D]Right upper quadrant pain
	R090K00	[D]Left upper quadrant pain
	R090L00	[D]Left lower quadrant pain
	R090M00	[D]Right lower quadrant pain
	R090N00	[D]Nonspecific abdominal pain
	R090P00	[D]Functional abdominal pain syndrome
	R090y00	[D]Other specified abdominal pain
	R090z00	[D]Abdominal pain NOS
	Ryu1000	[X]Pain localized to other parts of lower abdomen
	Ryu1100	[X]Other and unspecified abdominal pain
FC test	47J..00	Faecal calprotectin content
	47J0.00	Faecal calprotectin test indeterminate
	47J1.00	Faecal calprotectin test invalid
	47J2.00	Faecal calprotectin test positive
	47J3.00	Faecal calprotectin test negative
	4Q3J.00	Calprotectin level
**Variable**	**Drug code**	**Description**
Mesalazine	53913979	Mesalazine 800mg gastro-resistant tablets
	88517998	Mesalazine 400mg gastro-resistant tablets
	93624997	Mesalazine 250mg modified release tablets
	99583998	Mesalazine 250mg modified-release tablet
	54552979	Mesalazine 400mg gastro-resistant tablets
	55164978	Mesalazine 4g modified-release granules sachets sugar free
	55165978	Mesalazine 4g modified-release granules sachets sugar free
	58800979	Mesalazine 1.2g gastro-resistant modified-release tablets
	60584979	Mesalazine 3g gastro-resistant modified-release granules sachets sugar free
	60585979	Mesalazine 3g gastro-resistant modified-release granules sachets sugar free
	64868979	Mesalazine 2g modified-release granules sachets sugar free
	76424978	Mesalazine 1g modified-release tablets
	79867978	Mesalazine 400mg gastro-resistant tablets
	80928998	Mesalazine 3g gastro-resistant modified-release granules sachets sugar free

	80929998	Mesalazine 3g gastro-resistant modified-release granules sachets sugar free
	81193998	Mesalazine 1g modified-release tablets
	81194998	Mesalazine 1g modified-release tablets
	81683998	Mesalazine 1g suppositories
	81689998	Mesalazine 500mg gastro-resistant tablets
	81690998	Mesalazine 500mg gastro-resistant tablets
	81772998	Mesalazine 800mg gastro-resistant tablets
	81868998	Mesalazine 400mg gastro-resistant tablets
	83503998	Mesalazine 1.5g gastro-resistant modified-release granules sachets sugar free
	83504998	Mesalazine 1.5g gastro-resistant modified-release granules sachets sugar free
	83743998	Mesalazine 2g modified-release granules sachets sugar free
	83987998	Mesalazine 2g modified-release granules sachets sugar free
	84059998	Mesalazine 1g gastro-resistant modified-release granules sachets sugar free
	84209998	Mesalazine 800mg gastro-resistant tablets
	84290998	Mesalazine 1.2g gastro-resistant modified-release tablets
	84291998	Mesalazine 1.2g gastro-resistant modified-release tablets
	85560998	Mesalazine 800mg gastro-resistant tablets
	87761998	Mesalazine 400mg gastro-resistant tablets
	87909998	Mesalazine 1g gastro-resistant modified-release granules sachets sugar free
	87910998	Mesalazine 500mg gastro-resistant modified-release granules sachets sugar free
	87911998	Mesalazine 500mg gastro-resistant modified-release granules sachets sugar free
	89992997	Mesalazine 2g/59ml enema
	89992998	Mesalazine 500mg suppositories
	92346998	Mesalazine 1g/application foam enema
	92347998	Mesalazine 400mg gastro-resistant tablets
	92764997	Mesalazine 1g modified-release granules sachets sugar free
	92764998	Mesalazine 500mg modified-release tablets
	93623996	Mesalazine 250mg suppositories
	93623997	Mesalazine 1g/application foam enema
	93623998	Mesalazine 1g/100ml enema
	93624996	Mesalazine 1g suppositories
	93624998	Mesalazine 1g/100ml enema
	93728992	Mesalazine 500mg modified-release tablets
	94564992	Mesalazine 500mg modified-release tablets
	95041990	Mesalazine 400mg gastro-resistant tablets
	95888997	Mesalazine 250mg gastro-resistant tablets
	95888998	Mesalazine 400mg gastro-resistant tablets
	96608996	Mesalazine 2g/59ml enema
	96608997	Mesalazine 1g suppositories
	96608998	Mesalazine 500mg suppositories
	96659996	Mesalazine 1g/application foam enema
	96659997	Mesalazine 500mg suppositories

	96659998	Mesalazine 250mg suppositories
	96883990	Mesalazine 400mg gastro-resistant tablets
	96916992	Mesalazine 500mg modified-release tablets
	97381998	Mesalazine 400mg gastro-resistant tablets
	97764998	Mesalazine 250mg gastro-resistant tablets
	98001992	Mesalazine 250mg gastro-resistant tablets
	99486979	Mesalazine 400mg gastro-resistant tablets
	99487979	Mesalazine 400mg gastro-resistant tablets
	99488979	Mesalazine 400mg gastro-resistant tablets
	99490979	Mesalazine 1g modified-release granules sachets sugar free
	99492979	Mesalazine 1g suppositories
	99494979	Mesalazine 1g suppositories
	99495979	Mesalazine 500mg modified-release tablets
	99498979	Mesalazine 500mg modified-release tablets
	99583996	Mesalazine 1g gastro-resistant modified-release granules sachets sugar free
	99583997	Mesalazine 500mg modified-release tablets
Olsalazine	92400998	Olsalazine 500mg tablets
	92401998	Olsalazine 250mg capsules
	94437997	Olsalazine 500mg tablets
	94437998	Olsalazine 250mg capsules
	94438997	Olsalazine 500mg tablets
	94438998	Olsalazine 250mg capsules
Balsalazide	88489998	Balsalazide 750mg capsules
	88492998	Balsalazide 750mg capsules
Sulfasalazine	89598997	Sulfasalazine 3g/100ml retention enema
	89598998	Sulfasalazine 500mg suppositories
	89604997	Sulfasalazine 3g/100ml retention enema
	89604998	Sulfasalazine 500mg suppositories
	95256996	Sulfasalazine 3g/100ml enema
	95256997	Sulfasalazine 500mg suppositories
	97281996	Sulfasalazine 3g/100ml retention enema
	97281997	Sulfasalazine 500mg suppositories
Beclometasone	86941998	Beclometasone 5mg gastro-resistant modified-release tablets
	86942998	Beclometasone 5mg gastro-resistant modified-release tablets
Budenoside	50988978	Budesonide 9mg modified-release tablets
	50989978	Budesonide 9mg modified-release tablets
	60586979	Budesonide 9mg gastro-resistant granules sachets
	60587979	Budesonide 9mg gastro-resistant granules sachets
	80931998	Budesonide 9mg gastro-resistant granules sachets
	80932998	Budesonide 9mg gastro-resistant granules sachets
	84636998	Budesonide 2mg foam enema
	84637998	Budesonide 2mg foam enema
	89238998	Budesonide 2mg/100ml enema
	89239998	Budesonide 2mg/100ml enema

	94125992	Cortenema enema ml liq
Prednisolone	60097979	Prednisolone 20mg/application foam enema
	84741998	Prednisolone 40mg/100ml enema
	90310979	Prednisolone 20mg/application foam enema
	93706998	Prednisolone sodium phosphate 5mg suppositories
	94336997	Prednisolone sodium phosphate 5mg suppositories
	94336998	Prednisolone rectal ointment
	94451998	Prednisolone 20mg/application foam enema
	94452998	Prednisolone 20mg/application foam enema
	94468998	Prednisolone 20mg/100ml enema standard tube
	98370998	Prednisolone sodium phosphate 5mg suppositories
	98371998	Prednisolone 20mg/100ml enema standard tube
	99227997	Prednisolone 20mg/100ml enema standard tube
	99227998	Prednisolone 20mg/100ml enema standard tube
	99388979	Prednisolone 20mg/100ml enema standard tube
	99389979	Prednisolone 20mg/100ml enema standard tube
	89284997	Cinchocaine 1mg / Prednisolone hexanoate 1.3mg suppositories

Supplementary Table 2 Patient characteristics with FC tests included and excluded from the test accuracy study

	Included FC tests (N=5970)	Excluded FC tests (N=9370)	p-value
Age (mean (SD)) at FC test date, range	44.07 (16.23), 18-101	43.42 (16.54), 18-97	0.01594
Sex (female (%))	3828 (64.1)	5963 (63.6)	0.5452
BMI (mean (SD))	26.86 (6.25)	26.93 (6.22)	0.5285
Not reported n (%)	656 (10.99)	1061 (11.32)	0.521
Ethnicity n (%)			
White	3441 (57.64)	5507 (58.77)	0.1647
Asian	93 (1.56)	249 (2.66)	<0.0001
Black	63 (1.06)	135 (1.44)	0.0392
Mixed	24 (0.40)	64 (0.68)	0.0246
Other	33 (0.55)	55 (0.59)	0.7844
Not reported	2316 (38.79)	3360 (35.86)	0.0002
Townsend score n (%)			
1 (least deprived)	988 (16.55)	1936 (20.66)	<0.0001
2	1101 (18.44)	1691 (18.05)	0.5362
3	1140 (19.10)	1720 (18.36)	0.2518
4	1135 (19.01)	1666 (17.78)	0.0542
5 (most deprived)	1062 (17.79)	1149 (12.26)	<0.0001
Missing	544 (9.11)	1208 (12.89)	<0.0001
Country (n) (%)			
England	3748 (62.78)	7073 (75.49)	<0.0001
Northern Ireland	16 (0.27)	566 (6.04)	<0.0001
Scotland	1916 (32.09)	1007 (10.75)	<0.0001
Wales	290 (4.86)	724 (7.73)	<0.0001
CRC ever recorded n (%)	31 (0.52)	45 (0.48)	0.7373
IBS ever recorded n (%)	1897 (31.78)	2649 (28.27)	<0.0001
IBD ever recorded n (%)	208 (3.48)	208 (2.22)	<0.0001
IBD + IBS recorded n (%)	78 (1.31)	63 (0.67)	<0.0001
IBD + CRC recorded n (%)	2 (0.03)	0 (0)	0.0764
No diagnosis recorded n (%)	3754 (62.88)	6405 (68.36)	<0.0001

There were regional differences, with more FC tests excluded from Wales and Northern Ireland and fewer excluded from Scotland and England. There were also more excluded from the least deprived group according to Townsend Score, and fewer excluded from the most deprived group.

Supplementary Table 3 Test accuracy measures of FC testing at threshold of 100µg/g compared to 50µg/g

Threshold	N	TP	FP	FN	TN	Sensitivity, % (95% CI)	Specificity, % (95% CI)	PPV, % (95% CI)	NPV, % (95% CI)
50µg/g	5970	195	2219	15	3541	92.9 (88.6 to 95.6)	61.5 (60.2 to 62.7)	8.1 (7.1 to 9.2)	99.6 (99.3 to 99.7)
100µg/g	5970	181	1344	29	4416	86.2 (80.9 to 90.2)	76.7 (75.6 to 77.7)	11.9 (10.3 to 13.6)	99.3 (99.1 to 99.5)
250µg/g	5970	130	563	80	5197	61.9 (55.2 to 68.2)	90.2 (89.4 to 91)	18.8 (16 to 21.8)	98.5 (98.1 to 98.8)

N sample size, TP true positives, FP false positives, FN false negatives, TN true negatives, PPV positive predictive value, NPV negative predictive value, CI confidence interval
