# Supplementary material for: Test accuracy of faecal calprotectin for inflammatory bowel disease in UK primary care: a retrospective cohort study of the THIN data
Source: BMJ Open. 2021 Feb 22;11(2):e044177. doi: 10.1136/bmjopen-2020-044177 (PMC7903095; doi:10.1136/bmjopen-2020-044177)
Supplement: Author's manuscript [file bmjopen-2020-044177.draft_revisions.pdf]

BMJ Open is committed to open peer review. As part of this commitment we make the peer review history of every article we publish publicly available.

When an article is published we post the peer reviewers' comments and the authors' responses online. We also post the versions of the paper that were used during peer review. These are the versions that the peer review comments apply to.

The versions of the paper that follow are the versions that were submitted during the peer review process. They are not the versions of record or the final published versions. They should not be cited or distributed as the published version of this manuscript.

BMJ Open is an open access journal and the full, final, typeset and author-corrected version of record of the manuscript is available on our site with no access controls, subscription charges or pay-per-view fees (http://bmjopen.bmj.com).

If you have any questions on BMJ Open's open peer review process please email info.bmjopen@bmj.com

BMJ Open

Test Accuracy of Faecal Calprotectin for Inflammatory Bowel Disease in UK Primary Care: Retrospective Cohort Study of the IMRD-UK data

Journal:	*BMJ Open*
Manuscript ID	bmjopen-2020-044177
Article Type:	Original research
Date Submitted by the Author:	25-Aug-2020
Complete List of Authors:	Freeman, Karoline; University of Warwick, Warwick Medical School; University of Warwick<br>Taylor-Phillips, Sian; University of Warwick, Warwick Medical School<br>Willis, Brian; University of Birmingham, Institute of Applied Health Research<br>Ryan, Ronan; University of Birmingham, Institute of Applied Health Research<br>Clarke, Aileen; University of Warwick, Division of Health Sciences
Keywords:	Inflammatory bowel disease < GASTROENTEROLOGY, PRIMARY CARE, GASTROENTEROLOGY

SCHOLARONE™
Manuscripts

I, the Submitting Author has the right to grant and does grant on behalf of all authors of the Work (as defined in the below author licence), an exclusive licence and/or a non-exclusive licence for contributions from authors who are: i) UK Crown employees; ii) where BMJ has agreed a CC-BY licence shall apply, and/or iii) in accordance with the terms applicable for US Federal Government officers or employees acting as part of their official duties; on a worldwide, perpetual, irrevocable, royalty-free basis to BMJ Publishing Group Ltd ("BMJ") its licensees and where the relevant Journal is co-owned by BMJ to the co-owners of the Journal, to publish the Work in this journal and any other BMJ products and to exploit all rights, as set out in our licence.

The Submitting Author accepts and understands that any supply made under these terms is made by BMJ to the Submitting Author unless you are acting as an employee on behalf of your employer or a postgraduate student of an affiliated institution which is paying any applicable article publishing charge ("APC") for Open Access articles. Where the Submitting Author wishes to make the Work available on an Open Access basis (and intends to pay the relevant APC), the terms of reuse of such Open Access shall be governed by a Creative Commons licence – details of these licences and which Creative Commons licence will apply to this Work are set out in our licence referred to above.

Other than as permitted in any relevant BMJ Author's Self Archiving Policies, I confirm this Work has not been accepted for publication elsewhere, is not being considered for publication elsewhere and does not duplicate material already published. I confirm all authors consent to publication of this Work and authorise the granting of this licence.

For peer review only

Test Accuracy of Faecal Calprotectin for Inflammatory Bowel Disease in UK Primary Care: Retrospective Cohort Study of the IMRD-UK data

Karoline Freeman (0000-0002-9963-2918), Sian Taylor-Phillips, Brian H Willis, Ronan Ryan, Aileen Clarke

Karoline Freeman NIHR Doctoral Research Fellow, Division of Health Sciences, University of Warwick, Coventry, CV4 7AL, UK

Sian Taylor-Phillips Professor of Screening and Test Evaluation, Division of Health Sciences, University of Warwick, Coventry, CV4 7AL, UK

Brian H Willis MRC Clinician Scientist, Institute of Applied Health Research, University of Birmingham, Birmingham, B15 2TT, UK

Ronan Ryan Research Fellow, Institute of Applied Health Research, University of Birmingham, Birmingham, B15 2TT, UK

Aileen Clarke Professor of Public Health & Health Services Research, Division of Health Sciences, University of Warwick, Coventry, CV4 7AL, UK

Correspondence to:
Karoline Freeman: k.freeman@warwick.ac.uk

Words: 3797

For peer review only

ABSTRACT

Objective: To estimate the test accuracy of faecal calprotectin (FC) for inflammatory bowel disease (IBD) in the primary care setting using routine electronic health records.

Design: Retrospective cohort test accuracy study

Setting: UK primary care

Participants: 5970 patients (≥18 years) without a previous IBD diagnosis and with a first FC test between 1 January 2006 and 31 December 2016. We excluded multiple tests, tests without numeric results, with missing units or units other than µg/g.

Intervention: FC testing for the diagnosis of IBD. Disease status was confirmed by a recorded diagnostic code and/or a drug code of an IBD specific medication at three time points after the FC test date.

Main outcome measures: Sensitivity, specificity, positive and negative predictive values for the differential of IBD versus non-IBD and IBD versus IBS at the 50µg/g and 100µg/g thresholds.

Results: 5970 patients met the inclusion criteria and had at least 6 months of follow-up data after FC testing. 1897 had an IBS diagnosis, 208 had an IBD diagnosis, 31 had a colorectal cancer diagnosis, 80 had more than one diagnosis and 3754 had no subsequent diagnosis. Sensitivity, specificity, positive and negative predictive values were 92.9% (88.6% to 95.6%), 61.5% (60.2% to 62.7%), 8.1% (7.1% to 9.2%) and 99.6% (99.3% to 99.7%), respectively at the threshold of 50µg/g. Raising the threshold to 100µg/g missed less than 7% additional IBD cases. Longer follow-up had no effect on test accuracy. Overall, uncertainty was greater for specificity than sensitivity. General practitioners' referral decisions did not follow the anticipated clinical pathways in national guidance.

Conclusions: GPs can be confident in excluding IBD on the basis of a negative FC test in a population with low pre-test risk but should interpret a positive test with caution. The applicability of national guidance to general practice needs to be improved.

For peer review only - http://bmjopen.bmj.com/site/about/guidelines.xhtml

Strengths and limitations of this study

- The sample size in our study is likely to be larger than the usual cross-sectional studies used to evaluate a test, which is useful for IBD which has a low prevalence.
- We applied strict eligibility criteria to ensure the reliability of reported test accuracy measures and explored the effects of different follow-up times on the definition of IBD disease.
- We used a very comprehensive and sensitive list of Read codes and drug codes (48 codes) for the identification of IBD.
- The exclusion of close to half of the FC tests without a numerical test result raises concerns over the generalisability of the findings, however, findings are in line with published estimates.
- A coded IBD diagnosis or prescription is a proxy reference standard for a composite of different reference tests used in clinical practice reflecting a pragmatic approach to test accuracy.

For peer review only

INTRODUCTION

Inflammatory bowel disease is a chronic, progressive disorder caused by inflammation of the gastrointestinal tract. It often presents with non-specific symptoms such as abdominal pain and diarrhoea making it difficult to differentiate from irritable bowel syndrome (IBS), which is far more prevalent in the primary care setting. This also affects the numbers and composition of referrals to secondary care - with a higher proportion of patients being diagnosed in secondary care with IBS than is desirable. It is against this background that the non-invasive faecal calprotectin test has been introduced as a test for IBD in primary care in the UK. Faecal calprotectin (FC) is a small calcium-binding protein of the S100 family which is predominantly derived from neutrophils during an immune response. It is associated with inflammation and can be quantified in stool samples.[1] Therefore, it is more specific than serum inflammatory markers in the detection of gastrointestinal inflammation.[1]

The FC test is recommended by gastroenterological societies across the globe for its usefulness in the diagnosis of IBD.[2-5] However, there is no clear guidance on which settings it is considered appropriate. The National Institute for Health and Care Excellence (NICE) recommends the use of FC testing in UK primary care for the differential diagnosis of IBD and IBS when a referral is being considered and colorectal cancer is not suspected.[6] A study of routine data from an American health plans database of FC testing for chronic gastrointestinal symptoms in primary care suggests that it is used in routine general practice in at least one other country.[7] The NICE recommendations were based on an evaluation of secondary care test accuracy studies and assumptions that referral decisions would follow FC test results. This resulted in a slow uptake and inconsistent use of FC testing in primary care.[8] While primary care studies exist, we still lack compelling evidence on the test accuracy of FC testing in primary care for the detection of IBD. This is because the primary care evidence is based on small, heterogeneous studies that vary in clinical questions, FC thresholds and definitions of disease.[9] They report a range of sensitivities and specificities with wide confidence intervals and leave uncertainty over which threshold (50 or 100µg/g) should be adopted in primary care. To determine the utility of FC testing in primary care, a pragmatic study is required that both captures the differential diagnosis between IBD and IBS and has a patient population that is representative of primary care.

Thus, our primary aim was to determine the diagnostic value of FC for IBD in primary care by estimating the sensitivity, specificity and predictive values for two thresholds using routine UK primary care data from the IQVIA Medical Research Database (IMRD)-UK.

For peer review only - http://bmjopen.bmj.com/site/about/guidelines.xhtml

METHODS

Data source

We undertook a test accuracy study of FC testing for IBD using data from the IMRD-UK (formerly known as The Health Improvement Network (THIN)). IMRD-UK is a UK longitudinal primary care database of over 14 million patients from more than 670 GP practices covering about 6% of the UK population in 2015.[10] It has been found to be broadly representative of the UK population.[11] Patients' medical diagnoses, symptoms, laboratory tests, referrals and prescriptions are available as clinical codes (e.g. Read codes). The study was approved by the Independent Scientific Advisory Committee (protocol number 17THIN089).

Study design and population

This retrospective cohort study included patients (≥18 years) who had no previous IBD diagnosis and who had their first FC test between 1 January 2006 and 31 December 2016. Eligible patients had also contributed at least 12 months of data to IMRD-UK before their study start date. FC tests were identified using all six Read codes available to GPs to record an FC test.

Classification of FC test results

FC tests with a numeric result >50μg/g and >100μg/g were considered positive in two separate analyses. We included a post-hoc analysis at the threshold of 250μg/g in light of the latest recommendations of a pathway using two cut-offs.[8, 12] FC tests without results, with invalid results, qualitative results only, missing units of measurement, or units of measurement other than μg/g were excluded from the analysis.

Definition of disease

IBD was defined as a record of a Read code for ulcerative colitis, Crohn's disease, indeterminate colitis or microscopic colitis. Read code lists were adapted from those used in previous literature.[13-15] This was supplemented with the record of an IBD specific prescription to increase the sensitivity of the algorithm. IBS and colorectal cancer (CRC) were defined by any clinical code that suggested a definitive diagnosis. Abdominal symptoms were not considered sufficiently specific for an IBS diagnosis. We excluded benign and precancerous stages of CRC. In three different analyses a diagnosis was considered when it was recorded within 6 months, 12 months or 24 months of the FC test date to explore the impact of late

For peer review only - http://bmjopen.bmj.com/site/about/guidelines.xhtml

diagnoses in patients with sufficient follow-up after FC testing. We considered the following symptoms to assess eligibility for testing: diarrhoea, abdominal pain, constipation, bloating and change in bowel habit. See Supplementary Table 1 for final code lists of these variables.

Definition of referral

Our definition of IBD was based on the assumption that IBD diagnoses were only recorded following confirmatory testing in secondary care. We explored this assumption by investigating FC testing in the referred population only. We considered a referral to any speciality that was made within six weeks of the index FC test to be a referral in response to FC testing.

Statistical analysis

Data management and analysis were undertaken in R version 3.6.1 (Vienna, Austria).[16] The main outcomes of the analysis were sensitivity, specificity, and positive and negative predictive values. These were calculated in two ways: 1) Patients with a recorded IBD diagnosis were classed as disease positive and those without an IBD diagnosis (IBS, CRC, alternative diagnosis, no diagnosis) were classed as disease negative. (2) Patients with an IBD diagnosis were classed as disease positive and only those with an IBS diagnosis were classed as disease negative.

We used receiver operating characteristic (ROC) curves to display the accuracy of the FC test at test positive thresholds between 33 and 300μg/g. This allowed the inclusion of results recorded as >300μg/g and <33μg/g. ROC curves for different follow-up times were compared using Venkatraman's method for two unpaired ROC curves in the absence of a feasible paired comparison because data were of different sizes.[17]

We computed proportions of patients referred with a true positive (TP), false positive (FP), false negative (FN) and true negative (TN) FC test result in order to investigate the effect the FC test results have on patient management. If the test results had no impact on referral, the proportions across groups were expected to be similar. If GPs act on the test result (refer positives and manage negatives), the proportion of patients referred with TP and FP results was expected to be 1) close to one; 2) equal; and 3) greater than those for FN and TN results (both close to 0). The proportions of referred patients were compared using the two proportions z-test.

For peer review only - http://bmjopen.bmj.com/site/about/guidelines.xhtml

Sensitivity analyses were conducted in order to investigate the effects different assumptions have on the test accuracy estimates. Specifically, we investigated: including all units of measurements; including qualitative results in addition to quantitative results; defining IBD by Read code only; and confining analyses to only those patients referred to secondary care. We also explored the effects of excluding patients not eligible for the FC testing as according to national guidance (patients with an age >50 years or symptoms for <6 weeks).

Patient involvement

A patient advisory group was actively involved in the funding application for the project. The group provided a patient perspective in the design of the study and the interpretation of study findings.

RESULTS

17,466 first time FC tests in a total of 6,965,853 adult primary care patients were recorded in the study period, and 5,970 were included in the analysis (Figure 1). 1,987 (32%) had an IBS diagnosis, 208 (3.5%) an IBD diagnosis, 31 (0.5%) a colorectal cancer (CRC) diagnosis, 78 (1%) had an IBD and an IBS diagnosis, 2 had an IBD and a CRC diagnosis and 3,754 (63%) had no diagnosis recorded (see online Supplementary Table 2). 1,434/5970 (24%) patients had an IBS diagnosis recorded at the time of FC testing. The proportion of patients with an IBD or IBS diagnosis were slightly higher in the included than the excluded group (see online Supplementary Table 2).

++ Figure 1 ++

Test accuracy of FC testing for IBD

In 5,970 patients with at least 6 months follow-up available after the FC test date (Figure 1), the sensitivity of the FC test in detecting IBD at a threshold of 50µg/g was 92.9% (95% CI 88.6% to 95.6%). Specificity was 61.5% (95% 60.2% to 62.7%). The positive predictive value was low at 8.1% (95% CI 7.1% to 9.2%) while the negative predictive value was high (99.6% [95% CI 99.3% to 99.7%]). The inclusion of IBD cases that were recorded up to 12 (analysis of 4793 FC tested patients) and 24 months (2662 patients) following an FC test had no significant effect on the measures of test accuracy (Table 1). Small changes observed in the point estimates may be due to the different population size and the different make-up of the study population. 87% (2,096/2,414) of patients with a positive FC test result had no recorded diagnosis of IBD, IBS or CRC six months after the FC test date. Of these 501 had a previous diagnosis of IBS or CRC. At the end of the study period there were still

For peer review only - http://bmjopen.bmj.com/site/about/guidelines.xhtml

1,470 (61%) patients with a positive test but without a diagnosis of interest. These patients could have either have had a missed diagnosis, no diagnosis, or an alternative diagnosis, which was not included in our study. For patients with a negative FC test 92% (3,277/3,556) had none of the three diagnoses recorded at six months and 64% (2,284/3,556) had none of the three diagnoses at any time after FC testing.

Table 1 Test accuracy measures of FC testing at threshold of 50µg/g

**Time***	**N**	**TP**	**FP**	**FN**	**TN**	**Sensitivity, % (95% CI)**	**Specificity, % (95% CI)**	**PPV, % (95% CI)**	**NPV, % (95% CI)**
6 months	5970	195	2219	15	3541	92.9 (88.6 to 95.6)	61.5 (60.2 to 62.7)	8.1 (7.1 to 9.2)	99.6 (99.3 to 99.7)
12 months	4793	198	1790	16	2789	92.5 (88.2 to 95.3)	60.9 (59.5 to 62.3)	10 (8.7 to 11.4)	99.4 (99.1 to 99.6)
24 months	2662	129	1067	11	1455	92.1 (86.5 to 95.6)	57.7 (55.8 to 59.6)	10.8 (9.2 to 12.7)	99.2 (98.7 to 99.6)

*Time following FC testing to consider a record of IBD
N sample size, TP true positives, FP false positives, FN false negatives, TN true negatives, PPV positive predictive value, NPV negative predictive value, CI confidence interval

The ROC curves in Figure 2 describe the sensitivity and false positive rate (1- specificity) for the three different follow-up times at additional thresholds. The 50µg/g threshold prioritises sensitivity over specificity in all three analyses. There was generally good agreement between the three curves (E=0.0016758, p = 0.406 comparing 6 with 24 months follow-up).

++ Figure 2 ++

Increasing the FC thresholds

Figure 3 shows that an increase in the threshold from 50µg/g to 100µg/g affects the specificity more than the sensitivity (online Supplementary Table 3). Increasing the threshold reduces the number of false positives by 875 at the expense of missing an additional 14 cases of IBD in a population of 5,970 tested patients suggesting that the proportion of IBD cases missed due to this threshold difference is less than 7% (14/210). IBD cases missed were 31% (65/210) when we changed the threshold to 250µg/g (online Supplementary Table 3).

++ Figure 3 ++

Restricting the population to patients with IBD or IBS

Changing the definition of the non-diseased group from non-IBD to IBS reduced the number of false positives and true negatives. Consequently, the point estimates of specificity and

For peer review only - http://bmjopen.bmj.com/site/about/guidelines.xhtml

PPV increased by about 10% and 60%, respectively at the 50µg/g threshold (Table 2). However, the dataset was small (576 compared to 5,970) and, thus, increases the potential for sampling error. This may limit the clinical applicability of the findings.

Table 2 Test accuracy measures for FC testing at the threshold of 50µg/g for the detection of IBD recorded within 6 months of FC testing considering two different definitions of the non-diseased group

	N	TP	FP	FN	TN	Sensitivity, % (95% CI)	Specificity, % (95% CI)	PPV, % (95% CI)	NPV, % (95% CI)
IBD vs non-IBD	5970	195	2219	15	3541	92.9 (88.6 to 95.6)	61.5 (60.2 to 62.7)	8.1 (7.1 to 9.2)	99.6 (99.3 to 99.7)
IBD vs IBS	576	195	104	15	262	92.9 (88.6 to 95.6)	71.6 (66.8 to 76)	65.2 (59.7 to 70.4)	94.6 (91.3 to 96.7)

N sample size, TP true positive, FP false positive, FN false negative, TN true negative, CI confidence interval, PPV positive predictive value, NPV negative predictive value, IBD inflammatory bowel disease, IBS irritable bowel syndrome

Sensitivity analyses

Overall, the sensitivity analyses revealed greater uncertainty in the estimate of specificity than sensitivity (Table 3). Inclusion of numerical results reported in other units of measurement had no impact on estimates of test accuracy. The majority of qualitative results were positive at the 50µg/g threshold which may indicate preferential recording of positive test results using qualitative codes. In this sensitivity analysis the specificity was lower due to the great number of false positives. Only 569 patients tested had symptoms recorded that met the national guidance eligibility criteria. The recording of symptoms was not associated with the FC test result. Test accuracy measures in this subset did not differ from those estimated using the full dataset. Reclassifying 79 patients as IBD negative who had an IBD diagnosis affirmed by an IBD specific drug code only (Read code negative) had no impact on the test accuracy estimates. Finally, of the 5,970 FC tests, 52% resulted in a referral. The specificity of FC testing in this subgroup was lower because of the non-referral of mainly true negatives.

For peer review only - http://bmjopen.bmj.com/site/about/guidelines.xhtml

Table 3 Sensitivity analyses compared to primary analysis of FC testing for IBD vs non-IBD at a threshold of 50µg/g with IBD recorded within 6 months

Analysis	N	TP	FP	FN	TN	Sensitivity,% (95% CI)	Specificity, % (95% CI)	PPV, % (95% CI)	NPV, % (95% CI)
Primary analysis	5970	195	2219	15	3541	92.9 (88.6 to 95.6)	61.5 (60.2 to 62.7)	8.1 (7.1 to 9.2)	99.6 (99.3 to 99.7)
FC tests with any unit	6758	220	2481	17	4040	92.8 (88.8 to 95.5)	62 (60.8 to 63.1)	8.1 (7.2 to 9.2)	99.6 (99.3 to 99.7)
Qualitative results included*	7000	261	3177	15	3547	94.6 (91.2 to 96.7)	52.8 (51.6 to 53.9)	7.6 (6.8 to 8.5)	99.6 (99.3 to 99.7)
Eligible according to national guidelines**	569	20	194	1	354	95.2 (77.3 to 99.2)	64.6 (60.5 to 68.5)	9.3 (6.1 to 14)	99.7 (98.4 to 100)
IBD defined by Read code only	5970	148	2266	6	3550	96.1 (91.8 to 98.2)	61 (59.8 to 62.3)	6.1 (5.2 to 7.2)	99.8 (99.6 to 99.9)
Referred only*** (within 6 weeks)	3078	138	1330	11	1599	92.6 (87.3 to 95.8)	54.6 (52.8 to 56.4)	9.4 (8 to 11)	99.3 (98.8 to 99.6)

* with a record of an upper reference range of 50 or above

** Patients <50 years and abdominal symptoms for >6 weeks

*** Assumption that referred patients have high probability of disease verification by colonoscopy

TP true positives, FP false positives, FN false negatives, TN true negatives, PPV positive predictive value, NPV negative predictive value, FC faecal calprotectin

For peer review only

For peer review only - http://bmjopen.bmj.com/site/about/guidelines.xhtml

Impact of test results on referral decisions

Table 4 shows the classification of all included patients and those referred within 6 weeks of their FC test according to their test result and disease status. If GPs referred patients based on positive FC test results, they would have referred 2,414/5,970 (40.4%) included patients. This would have detected 195/210 (92.9%) of IBD cases. However, six weeks after FC testing 3,078/5,970 (51.6%) patients were referred, including 1,610/3,556 (45.3%) FC negative patients and only 149/210 (71.0%) of IBD cases. Therefore, the test result was not the single determining factor for or against a timely referral. Patients with true positive and false negative FC test results were equally likely to be referred for further tests (70.8% vs 73.3%, z=2, p= 0.8371). Both groups were referred more frequently than patients with false positive (59.9%) (z=3, p= 0.0028) or true negative results (45.3%) (z=7, p<0.0001). This may suggest that GPs used alternative cues (in the history, examination or other tests) which raised their suspicion of IBD. Overall, GP assessment plus FC testing may be worse than FC testing alone. Compared to FC testing alone, the number of patients with a true positive result was lower for GP assessment plus testing because GPs did not refer all test positive patients within six weeks. GPs referred some patients with a false negative FC test result on the test, but not enough to make up for not referring patients with true positive results on the test.

This interpretation assumes that referrals were recorded consistently for those with and without an IBD record and irrespective of FC test outcome. We interpret the findings with caution because we could not confirm the final classification of more than half of the tested patients and missing IBD cases cannot be excluded. Furthermore, all IBD patients would have been eventually referred according to the definition of an IBD diagnosis, which assumed that IBD diagnoses are only recorded following confirmation by a gastroenterologist and invasive testing. 61/210 (29.0%) IBD cases were either referred after 6 weeks or their referral was not recorded.

Table 4 Proportion of patients referred by test result and disease status

	**TP**	**FP**	**FN**	**TN**	**Total**
Classification of included patients in primary analysis based on FC test result n	195	2,219	15	3,541	5,970
Classification of FC tested patients with referral based on FC test result n	138	1,330	11	1,599	3,078
Proportion of patients in primary analysis referred, %	70.8	59.9	73.3	45.3	51.6

For peer review only - http://bmjopen.bmj.com/site/about/guidelines.xhtml

DISCUSSION

Study findings

After excluding FC tests with no result or an ambiguous result, the sensitivity of the FC test in detecting IBD was high (92.9% [95% CI 88.6% to 95.6%]) but the specificity was modest (61.5% [95% 60.2% to 62.7%]). Moreover, the sensitivity analyses demonstrated greater uncertainty in our estimates of the specificity. The high false positive rate may be irrelevant in clinical practice if the group represents alternative inflammatory conditions that warrant further assessment. However, a broader disease category in primary care including other organic conditions in addition to IBD resulted in sensitivity estimates ranging from 64% to 94%.[18-20] As the conditions may follow different pathways, interpretation of a test positive result would still be challenging. Restricting the group without IBD to those with a confirmed IBS record increased the specificity by 11 percentage points but has little meaning for clinical practice because it is an artificial patient population. Extending the follow-up time to allow for late recordings of IBD had no impact on sensitivity and specificity. The ROC analysis revealed that the recommended FC threshold of 50µg/g prioritises sensitivity over specificity.

Changing the threshold from 50µg/g to 100µg/g had a greater effect on the specificity than the sensitivity. For clinical practice this could translate into fewer referrals of non-IBD patients to gastroenterology at the expense of missing an additional nearly 7% of IBD cases. This interpretation is purely based on the logic of test results. Investigation of the proportion of referrals of patients with true positive, false positive, false negative and true negative test results showed that the actual management of tested patients may not clearly follow the expected behaviour of referring positives and managing test negative patients.

Strengths and limitations

One benefit of using routine data to estimate a test's accuracy is that the sample size is likely to be larger than the usual cross-sectional studies used to evaluate a test. This is particularly useful for target disorders such as IBD which have a low prevalence.[21] We applied strict eligibility criteria to ensure the reliability of reported test accuracy measures and we explored the effects of different follow-up times on the definition of IBD disease. In the case of FC testing in routine primary care, where it is not feasible to expose all tested patients to colonoscopy for disease verification, we were able to provide an answer to the question of the accuracy of the FC test as it is used in UK general practice. Our study shows that FC testing together with the general practitioner's decision-making is an effective tool to exclude IBD in test negative patients.

For peer review only - http://bmjopen.bmj.com/site/about/guidelines.xhtml

The study may have suffered from selection bias because the inclusion criterion was a recorded FC test rather than symptomatic patients.[22] However, we believe this to be minimal when considering the point estimates and confidence intervals of test accuracy measures in the sensitivity analysis of patients with eligible symptoms.

The exclusion of close to half of the FC tests without a numerical test result raises concerns over the generalisability of the findings because excluded tests were statistically different to included tests in some variables tested. However, the tests were highly powered because of the large dataset and the clinical significance of the differences is highly uncertain.

Although imputation methods are often used to mitigate against missing data these were not implemented for three reasons. Firstly, in observational data, observed variables are not sufficient to account for the differences between missing and observed values.[23] Secondly, for imputation methods to be meaningful the proportion of missing data should be small and the missing subpopulation should be sufficiently similar to the wider population neither of which could be confirmed. Thirdly, in order to build reliable imputation models, more meaningful variables are needed including severity of symptoms, assay type, referral to gastroenterology and information on disease verification method, none of which is available in the IMRD-UK database. Therefore, potential biases could only be addressed by sensitivity analyses.[23]

The proportion of patients without a diagnosis following testing was large and could be due to any of the following: missed IBD; unrecorded IBS; other conditions not considered as study variables; or diagnoses recorded as free text and thus not available to the study. Also the research literature was too heterogeneous to be able to estimate the prevalence of other conditions accurately. For example, across 5 studies[24-28] the number of conditions varied widely and the prevalence ranged from 4% (16/399)[24] to 61% (422/694).[27] We were unable to predict IBD using other variables, such as symptoms and co-morbidities, in the non-IBD group due to the lack of strong predictors of IBD among the study variables. Without any further information on the characteristics of the non-IBD cases their true disease status remains uncertain.

Our results are broadly in line with pooled estimates from a meta-analysis of 14 secondary and primary are studies,[9] which may suggest that the pooled test accuracy measures are applicable to the FC tested population in primary care. At the thresholds investigated, our sensitivity and specificity estimates are within the range of estimates from published primary care studies[24, 29, 30] However, previous primary care studies included small numbers of cases

For peer review only - http://bmjopen.bmj.com/site/about/guidelines.xhtml

while our study provides narrower confidence intervals around test accuracy estimates. Crucially, our study shows that the specificity of 94% (95% CI 73% to 99%) reported in the NICE guidance is not applicable to the primary care setting,[6] where the broader definition of the non-IBD group means that the specificity and the positive predictive value of FC testing is much lower. Therefore, the interpretation of a test positive result may be challenging. Furthermore, the higher number of patients with a false positive result may not achieve the reduction of referrals to colonoscopy as anticipated by NICE guidance.

Implications for clinical practice

This study indicates that FC testing, when used by GPs, does not miss many cases of IBD in a low prevalence setting (NPV 99.6% [95% CI 99.3% to 99.7%]). GPs could, therefore, use the test confidently in ruling out IBD if the test is negative. However, the test does not perform as well in identifying IBD as modelled by national guidance. The positive predictive value of the FC test in identifying IBD is low, as essentially FC is a marker of intestinal inflammation rather than a specific marker for IBD.

The data also suggested that the higher threshold of 100µg/g may be appropriate for routine primary care. This is under the understanding that the test is not used in isolation and the objective is to reduce the number of unnecessary colonoscopies. Missed IBD patients would be assumed to re-present and be re-considered for referral within an appropriate time frame. However, the latest FC pathway recommends two cut-offs (100µg/g and 250µg/g)[8, 12] with an intermediate test range for retesting. The additional IBD cases missed at initial testing with this strategy may be inappropriately high. In this study we have not considered the feasibility of this latest pathway but it is likely to be more difficult to interpret for general practitioners.

Our study identified a number of inconsistencies between the intended use by national guidelines and the actual use by general practitioners. Firstly, general practitioners tested an unexpectedly high proportion of IBS patients which may suggest that general practitioners use the test to reassure patients with IBS. Secondly, practitioners referred proportionately more patients with false negative results than false positive and true negative results. This suggests that practitioners use other cues for referral rather than test results alone. Thirdly, close to half of all negative FC tested patients were referred, which suggests that general practitioners and/or patients may have limited confidence in the test. The current guidelines may not be applicable to general practice and the modelled cost-effectiveness of FC testing in general practice may be overoptimistic. When developing national guidance models should more closely represent general practice, rather than simply extrapolating from secondary care. This study shows for the first time the benefits and shortcomings of the

For peer review only - http://bmjopen.bmj.com/site/about/guidelines.xhtml

performance of FC testing in a large dataset of primary care patients. It should help improve GPs' confidence in their diagnostic ability in this area and in test use and interpretation – especially where there is a negative test.

For peer review only

For peer review only - http://bmjopen.bmj.com/site/about/guidelines.xhtml

REFERENCES

1. Schroder O, Naumann M, Shastri Y, Povse N, Stein J. Prospective evaluation of faecal neutrophil-derived proteins in identifying intestinal inflammation: combination of parameters does not improve diagnostic accuracy of calprotectin. *Aliment Pharmacol Ther*. 2007;26(7):1035-42. doi: 10.1111/j.1365-2036.2007.03457.x.
2. Lichtenstein GR, Loftus EV, Isaacs KL, Regueiro MD, Gerson LB, Sands BE. ACG clinical guideline: management of Crohn's disease in adults. *Am J Gastroenterol*. 2018;113:481. doi: 10.1038/ajg.2018.27 https://www.nature.com/articles/ajg201827#supplementary-information.
3. The Gastroenterological Society of Australia. Australian guidelines for general practitioners and physicians: inflammatory bowel disease. 2017 [cited 08/10/2018]; Available from: http://cart.gesa.org.au/membes/files/Clinical%20Guidelines%20and%20Updates/2017_IBD_guidelines_DRAFT_for_consultation_20102017.pdf.
4. Preiß J, Bokemeyer B, Buhr H, Dignaß A, Häuser W, Hartmann F, Herrlinger K, Kaltz B, Kienle P, Kruis W, Kucharzik T, Langhorst J, Schreiber S, Siegmund B, Stallmach A, Stange E, Stein J, Hoffmann J. Updated German clinical practice guideline on "Diagnosis and treatment of Crohn's disease". 2014 [cited 08/10/2018]; Available from: https://www.dgvs.de/wp-content/uploads/2016/11/DGVS_Empfehlung_fuer_Diagnistik_und_Therapie_des_Morbus_Crohn.pdf.
5. Maaser C, Sturm A, Vavricka SR, Kucharzik T, Fiorino G, Annese V, Calabrese E, Baumgart DC, Bettenworth D, Borralho Nunes P, Burisch J, Castiglione F, Eliakim R, Ellul P, González-Lama Y, Gordon H, Halligan S, Katsanos K, Kopylov U, Kotze PG, Krustinš E, Laghi A, Limdi JK, Rieder F, Rimola J, Taylor SA, Tolan D, van Rheenen P, Verstockt B, Stoker J. ECCO-ESGAR Guideline for diagnostic assessment in IBD part 1: initial diagnosis, monitoring of known IBD, detection of complications. *J Crohns Colitis*. 2019;13(2):144-64. doi: 10.1093/ecco-jcc/jjy113.
6. National Institute for Health and Care Excellence. Faecal calprotectin diagnostic tests for inflammatory diseases of the bowel: NICE diagnostics guidance [DG11]. 2013 [cited 17/12/2015]; Available from: https://www.nice.org.uk/guidance/dg11.
7. Ramraj R, Garcia A, Mosen D, Waiwaiole L, Smith N. Utility of fecal calprotectin in evaluation of chronic gastrointestinal symptoms in primary care. *Clin Pediatr (Phila)*. 2018;57(9):1058-63. doi: 10.1177/0009922817744607.
8. CSO Faecal Calprotectin Working Group. Faecal calprotectin in primary care as a decision diagnostic for inflammatory bowel disease and irritable bowel syndrome. 2018 [cited 09/07/2018]; Available from: https://www.pcc-cic.org.uk/article/updated-guidance-help-gps-easily-diagnose-serious-bowel-conditions.
9. Freeman K, Willis BH, Fraser H, Taylor-Phillips S, Clarke A. Faecal calprotectin to detect inflammatory bowel disease: a systematic review and exploratory meta-analysis of test accuracy. *BMJ Open*. 2019;9(3):e027428-e. doi: 10.1136/bmjopen-2018-027428.
10. Kontopantelis E, Stevens RJ, Helms PJ, Edwards D, Doran T, Ashcroft DM. Spatial distribution of clinical computer systems in primary care in England in 2016 and implications for primary care electronic medical record databases: a cross-sectional population study. *BMJ Open*. 2018;8(2):e020738. doi: 10.1136/bmjopen-2017-020738.
11. Blak BT, Thompson M, Dattani H, Bourke A. Generalisability of The Health Improvement Network (THIN) database: demographics, chronic disease prevalence and mortality rates. *Inform Prim Care*. 2011;19(4):251-5. doi: 10.14236/jhi.v19i4.820.
12. Turvill J, Turnock D. Audit of the impact of the York faecal calprotectin care pathway on colonoscopy activity. *Frontline Gastroenterol*. 2019;0:1-5. doi: 10.1136/flgastro-2019-101315.
13. The University of Manchester. Clinical Codes Repository. [cited 16/04/2020]; Available from: https://clinicalcodes.rss.mhs.man.ac.uk/.

For peer review only - http://bmjopen.bmj.com/site/about/guidelines.xhtml

14. Harkness EF, Grant L, O'Brien SJ, Chew-Graham CA, Thompson DG. Using read codes to identify patients with irritable bowel syndrome in general practice: a database study. *BMC family practice*. 2013;14:183-. doi: 10.1186/1471-2296-14-183.
15. Lewis JD, Brensinger C, Bilker WB, Strom BL. Validity and completeness of the General Practice Research Database for studies of inflammatory bowel disease. *Pharmacoepidemiol Drug Saf*. 2002;11(3):211-8. doi: 10.1002/pds.698.
16. R Core Team. R: A language and environment for statistical computing. R Foundation for Statistical Computing, Vienna, Austria. [computer program]. 2017. Available from: https://www.R-project.org/.
17. Venkatraman ES. A permutation test to compare receiver operating characteristic curves. *Biometrics*. 2000;56(4):1134-8. doi: 10.1111/j.0006-341x.2000.01134.x.
18. Pavlidis P, Chedgy FJ, Tibble JA. Diagnostic accuracy and clinical application of faecal calprotectin in adult patients presenting with gastrointestinal symptoms in primary care. *Scand J Gastroenterol*. 2013;48(9):1048-54. doi: http://dx.doi.org/10.3109/00365521.2013.816771.
19. Turvill J, Turnock D, Holmes H, Jones A, McLaughlan E, Hilton V, Marriott S. Evaluation of the clinical and cost-effectiveness of the York Faecal Calprotectin Care Pathway. *Frontline Gastroenterol*. 2018;9:285-94. doi: 10.1136/flgastro-2018-100962.
20. Walker G, Moore L, Heerasing N, Hendy P, Perry M, McDonald T, Debenham T, Bethune R, Bewshea C, Hyde C, Heap G, Singh A, Calvert C, Kennedy N, Goodhand J, Ahmad T. Faecal calprotectin effectively excludes inflammatory bowel disease in 789 symptomatic young adults with/without alarm symptoms: a prospective UK primary care cohort study. *Aliment Pharmacol Ther*. 2018;47. doi: 10.1111/apt.14563.
21. Holtman GA, Berger MY, Burger H, Deeks JJ, Donner-Banzhoff N, Fanshawe TR, Koshiaris C, Leeflang MM, Oke JL, Perera R, Reitsma JB, Van den Bruel A. Development of practical recommendations for diagnostic accuracy studies in low-prevalence situations. *J Clin Epidemiol*. 2019;114:38-48. doi: https://doi.org/10.1016/j.jclinepi.2019.05.018.
22. Oostenbrink R, Moons KGM, Bleeker SE, Moll HA, Grobbee DE. Diagnostic research on routine care data: prospects and problems. *J Clin Epidemiol*. 2003;56(6):501-6. doi: https://doi.org/10.1016/S0895-4356(03)00080-5.
23. Sterne JAC, White IR, Carlin JB, Spratt M, Royston P, Kenward MG, Wood AM, Carpenter JR. Multiple imputation for missing data in epidemiological and clinical research: potential and pitfalls. *BMJ*. 2009;338:b2393. doi: 10.1136/bmj.b2393.
24. Conroy S, Hale MF, Cross SS, Swallow K, Sidhu RH, Sargur R, Lobo AJ. Unrestricted faecal calprotectin testing performs poorly in the diagnosis of inflammatory bowel disease in patients in primary care. *J Clin Pathol*. 2018;71:316-22. doi: http://dx.doi.org/10.1136/jclinpath-2017-204506.
25. Labaere D, Smismans A, Van Olmen A, Christiaens P, D'Haens G, Moons V, Cuyle PJ, Frans J, Bossuyt P. Comparison of six different calprotectin assays for the assessment of inflammatory bowel disease. *United European Gastroenterol J*. 2014;2(1):30-7. doi: https://dx.doi.org/10.1177/2050640613518201.
26. De Sloovere MM, De Smet D, Baert FJ, Debrabandere J, Vanpoucke HJ. Analytical and diagnostic performance of two automated fecal calprotectin immunoassays for detection of inflammatory bowel disease. *Clin Chem Lab Med*. 2017;55(9):1435-46. doi: https://dx.doi.org/10.1515/cclm-2016-0796.
27. Mowat C, Digby J, Strachan JA, Wilson R, Carey FA, Fraser CG, Steele RJ. Faecal haemoglobin and faecal calprotectin as indicators of bowel disease in patients presenting to primary care with bowel symptoms. *Gut*. 2016;65(9):1463-9. doi: https://dx.doi.org/10.1136/gutjnl-2015-309579.
28. Oyaert M, Trouve C, Baert F, De Smet D, Langlois M, Vanpoucke H. Comparison of two immunoassays for measurement of faecal calprotectin in detection of inflammatory bowel disease: (pre)-analytical and diagnostic performance characteristics. *Clin Chem Lab Med*. 2014;52(3):391-7. doi: https://dx.doi.org/10.1515/cclm-2013-0699.

For peer review only - http://bmjopen.bmj.com/site/about/guidelines.xhtml

29. Boyd A, Peplow C, Dhaliwal A, O'Rourke J, Davies J, Milestone A. Primary care faecal calprotectin pathway: a three year analysis. *Colorectal Disease*. 2016;18:27. doi: http://dx.doi.org/10.1111/codi.13444.
30. Hogberg C, Karling P, Rutegard J, Lilja M. Diagnosing colorectal cancer and inflammatory bowel disease in primary care: the usefulness of tests for faecal haemoglobin, faecal calprotectin, anaemia and iron deficiency. A prospective study. *Scand J Gastroenterol*. 2017;52(1):69-75. doi: https://dx.doi.org/10.1080/00365521.2016.1228120.

For peer review only

For peer review only - http://bmjopen.bmj.com/site/about/guidelines.xhtml

FIGURE LEGEND

Figure 1 Flow diagram of inclusion criteria of FC tested patients into test accuracy study

Figure 2 ROC curve of sensitivity and false positive rate (1-specificity) for thresholds 33-300μg/g for the clinical question IBD versus non-IBD for tests with at least 6 months (N=5,970), 12 months (N=4,793) and 24 months (N=2,662) follow-up available after the FC test date

Figure 3 Sensitivity and specificity at thresholds 33-300μg/g for IBD versus non-IBD and IBD recorded within 6 months following the FC test date (N=5,970)

For peer review only

Author contributions

KF, STP, BHW, RR and AC designed the study. RR extracted the data from IMRD-UK and created the datasets. KF, STP and BW carried out the analysis. KF, BHW, STP and AC contributed to the interpretation of the findings. KF drafted the manuscript. All authors critically revised the manuscript and approved the final version. KF takes responsibility for the integrity and accuracy of the data analysis. KF acts as guarantor. The corresponding author attests that all listed authors meet authorship criteria and that no others meeting the criteria have been omitted.

Acknowledgement

We would like to thank the patient advisory group of the project 'What is the role of faecal calprotectin testing in primary care" for their input.

Funding

KF is funded by a National Institute for Health Research (NIHR) DRF award (DRF-2016-09-038) for this research project. AC is supported by the National Institute for Health Research (NIHR) Applied Research Collaboration (ARC) West Midlands. This report presents independent research funded by the National Institute for Health Research (NIHR). The views expressed are those of the author and not necessarily those of the NHS, the NIHR or the Department of Health and Social Care. The funder had no role in the study design, data collection, data analysis and interpretation, writing of the report or the decision to submit for publication.

Competing interests

All authors have completed the Unified Competing Interest form (available on request from the corresponding author) and declare: KF is funded by the NIHR through a doctoral research fellowship. STP reports grants from the NIHR fellowship for Karoline Freemen. AC is supported by the NIHR ARC West Midlands initiative. BHW received grants from the Medical Research Council. RR has no conflicts to declare.

Data sharing statement

The IMRD-UK dataset cannot be shared under the data sharing agreement with the University of Birmingham on behalf of IQVIA.

Transparency

The lead author and manuscript's guarantor (KF) affirms that the manuscript is an honest, accurate and transparent account of the study being reported; that no important aspects of

For peer review only - http://bmjopen.bmj.com/site/about/guidelines.xhtml

For peer review only

the study have been omitted; and that any discrepancies from the study as planned have been explained.

Ethical approval

The study was approved by the Independent Scientific Advisory Committee (protocol number 17THIN089).

Dissemination declaration

We will disseminate the results to national charities of IBD and IBS patients.

Exclusive licence

The Corresponding Author has the right to grant on behalf of all authors and does grant on behalf of all authors, an exclusive licence (or non exclusive for government employees) on a worldwide basis to the BMJ Publishing Group Ltd to permit this article (if accepted) to be published in BMJ editions and any other BMJPGL products and sublicences such use and exploit all subsidiary rights, as set out in BMJ licence.

For peer review only

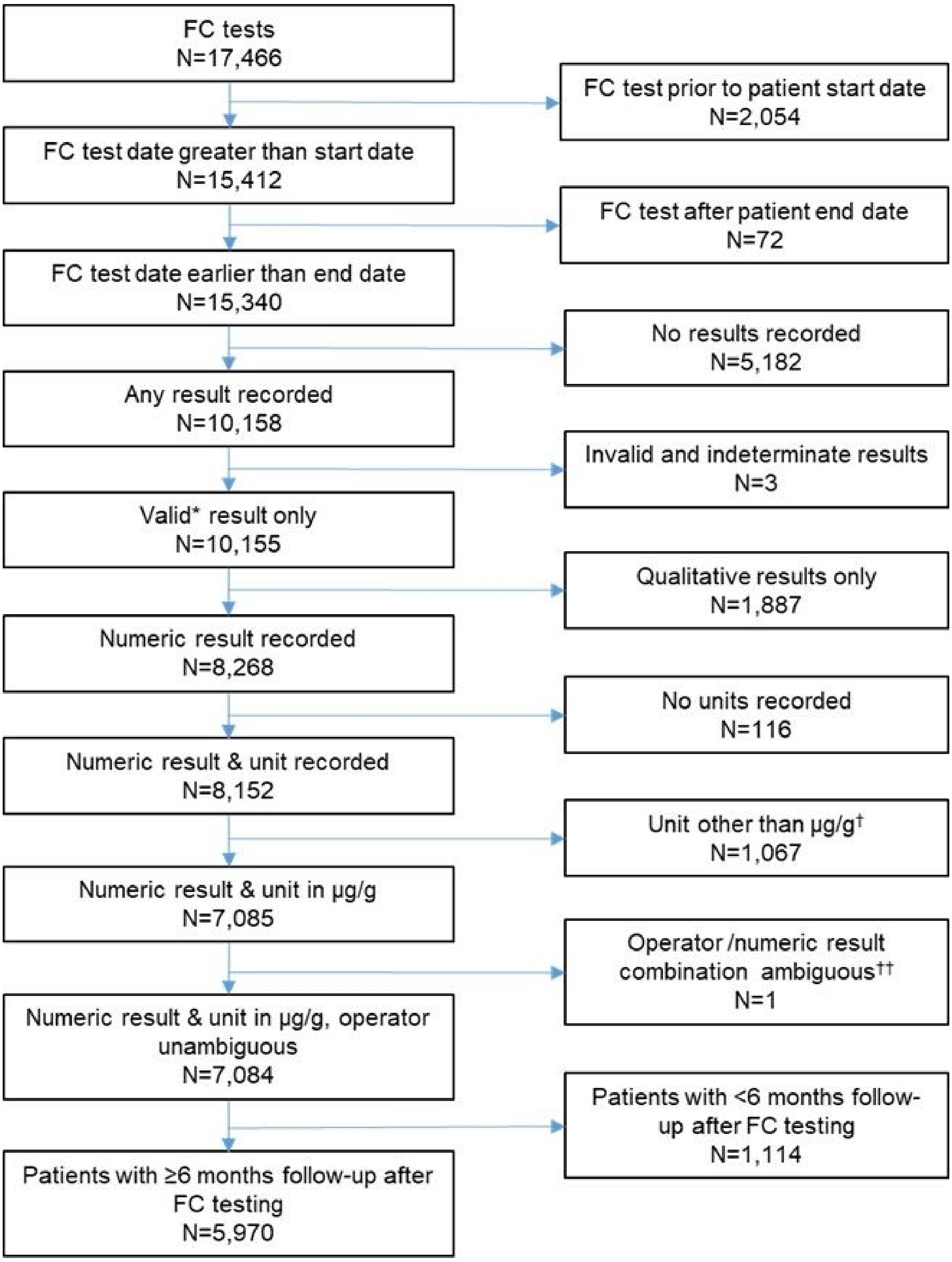

Figure 1 Flow diagram of inclusion criteria of FC tested patients into test accuracy study

*Results not coded invalid or indeterminate in IMRD-UK

†Fifteen different units of measurements were used to record FC levels. µg/g is the most commonly used in publications and laboratories.

††FC level of >30µg/g ambiguous at 50µg/g and 100µg/g thresholds

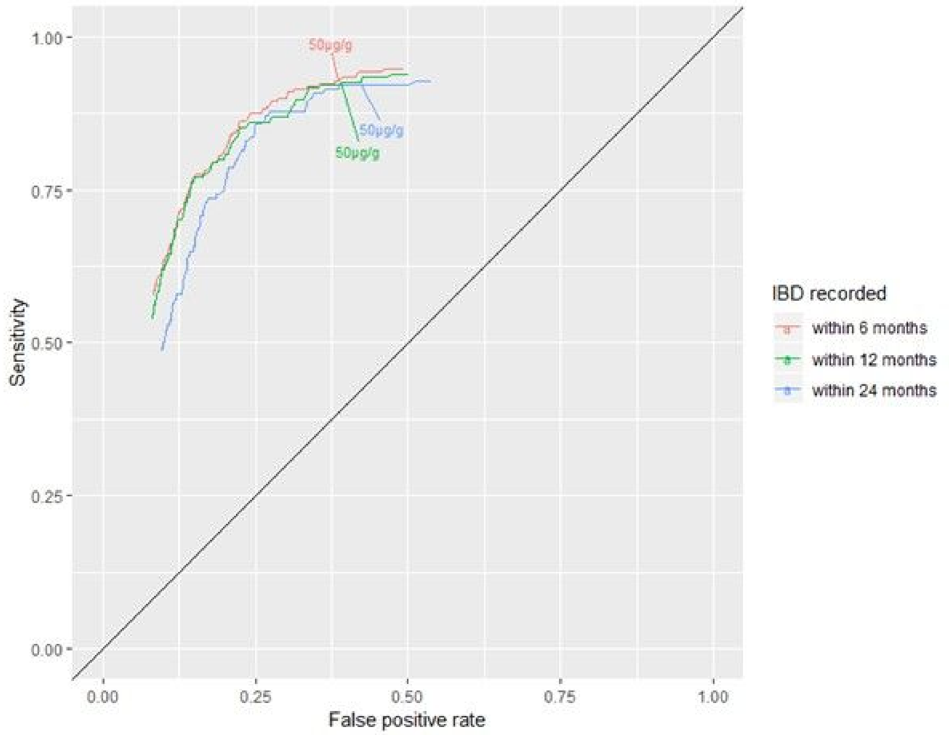

Figure 2 ROC curve of sensitivity and false positive rate (1-specificity) for thresholds 33-300µg/g for the clinical question IBD versus non-IBD for tests with at least 6 months (N=5,970), 12 months (N=4,793) and 24 months (N=2,662) follow-up available after the FC test date

For peer review only

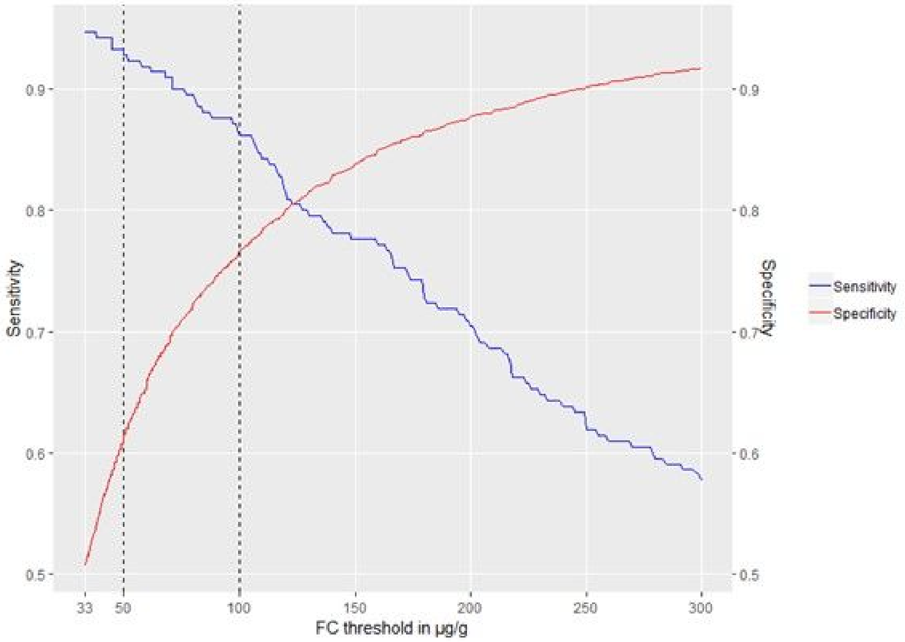

Figure 3 Sensitivity and specificity at thresholds 33-300µg/g for IBD versus non-IBD and IBD recorded within 6 months following the FC test date (N=5,970)

SUPPLEMENT

Supplementary Table 1 Final variable code lists

Variable	medcode	description
IBD	14C4.11	H/O: ulcerative colitis
	J08z900	Orofacial Crohn's disease
	J4...12	Inflammatory bowel disease
	J40..00	Regional enteritis - Crohn's disease
	J40..11	Crohn's disease
	J40..12	Granulomatous enteritis
	J400.00	Regional enteritis of the small bowel
	J400000	Regional enteritis of the duodenum
	J400100	Regional enteritis of the jejunum
	J400200	Crohn's disease of the terminal ileum
	J400300	Crohn's disease of the ileum unspecified
	J400400	Crohn's disease of the ileum NOS
	J400500	Exacerbation of Crohn's disease of small intestine
	J400z00	Crohn's disease of the small bowel NOS
	J401.00	Regional enteritis of the large bowel
	J401000	Regional enteritis of the colon
	J401100	Regional enteritis of the rectum
	J401200	Exacerbation of Crohn's disease of large intestine
	J401z00	Crohn's disease of the large bowel NOS
	J401z11	Crohn's colitis
	J402.00	Regional ileocolitis
	J40z.11	Crohn's disease NOS
	J41..12	Ulcerative colitis and/or proctitis
	J410.00	Ulcerative proctocolitis
	J410000	Ulcerative ileocolitis
	J410100	Ulcerative colitis
	J410200	Ulcerative rectosigmoiditis
	J410300	Ulcerative proctitis
	J410400	Exacerbation of ulcerative colitis
	J410z00	Ulcerative proctocolitis NOS
	J411.00	Ulcerative (chronic) enterocolitis
	J412.00	Ulcerative (chronic) ileocolitis
	J413.00	Ulcerative pancolitis
	J436.00	Microscopic colitis
	J436000	Collagenous colitis
	J436100	Lymphocytic colitis
	J438.00	Left sided colitis
	J4z3.00	Non-infective colitis NOS
	J4z4.00	Non-infective sigmoiditis NOS
	J4z6.00	Indeterminate colitis

For peer review only

	Jyu4000	[X]Other Crohn's disease
	Jyu4100	[X]Other ulcerative colitis
	N031000	Arthropathy in ulcerative colitis
	N031100	Arthropathy in Crohn's disease
	N045300	Juvenile arthritis in Crohn's disease
	N045400	Juvenile arthritis in ulcerative colitis
	ZR3S.00	Crohn's disease activity index
	ZR3S.11	CDAI - Crohn's disease activity index
IBS	14CF.00	History of irritable bowel syndrome
	8CA4a00	Education about FODMAP exclusion diet
	8CA4Z00	Dietary education for irritable bowel syndrome
	8Cm..00	Management of irritable bowel syndrome
	Eu45324	[X]Psychogenic IBS
	J521.00	Irritable colon - Irritable bowel syndrome
	J521.11	Irritable bowel syndrome
	J521.13	Spastic colon
	J521000	Irritable bowel syndrome with diarrhoea
	J521100	Irritable bowel syndrome characterised by constipation
	J521200	IBS characterised by alternating bowel habit
Colorectal cancer	68W2400	Bowel scope (flexible sigmoidoscopy) screen: cancer detected
	68W2500	Bowel scope (flexi-sig) screen: suspected cancer detected
	9Ow1.00	Bowel cancer detected by national screening programme
	B13..00	Malignant neoplasm of colon
	B130.00	Malignant neoplasm of hepatic flexure of colon
	B131.00	Malignant neoplasm of transverse colon
	B132.00	Malignant neoplasm of descending colon
	B133.00	Malignant neoplasm of sigmoid colon
	B134.00	Malignant neoplasm of caecum
	B134.11	Carcinoma of caecum
	B136.00	Malignant neoplasm of ascending colon
	B137.00	Malignant neoplasm of splenic flexure of colon
	B138.00	Malignant neoplasm, overlapping lesion of colon
	B139.00	Hereditary nonpolyposis colon cancer
	B13y.00	Malignant neoplasm of other specified sites of colon
	B13z.00	Malignant neoplasm of colon NOS
	B13z.11	Colonic cancer
	B14..00	Malignant neoplasm of rectum, rectosigmoid junction and anus
	B140.00	Malignant neoplasm of rectosigmoid junction
	B141.00	Malignant neoplasm of rectum
	B141.11	Carcinoma of rectum
	B141.12	Rectal carcinoma
	B142000	Malignant neoplasm of cloacogenic zone

For peer review only

	B14y.00	Malig neop other site rectum, rectosigmoid junction and anus
	B14z.00	Malignant neoplasm rectum,rectosigmoid junction and anus NOS
	B1z0.11	Cancer of bowel
	B575.00	Secondary malignant neoplasm of large intestine and rectum
	B575000	Secondary malignant neoplasm of colon
	B575100	Secondary malignant neoplasm of rectum
	B575z00	Secondary malig neop of large intestine or rectum NOS
	B902400	Neoplasm of uncertain behaviour of colon
	B902500	Neoplasm of uncertain behaviour of rectum
	BB5N.00	[M]Adenomatous and adenocarcinomatous polyps of colon
	BB5N.11	[M]Adenoma or or adenocarcinoma in polyposis coli
	BB5N100	[M]Adenocarcinoma in adenomatous polposis coli
	BB5Nz00	[M]Adenomatous or adenocarcinomatous polyps of the colon NOS
	ZV10017	[V]Personal history of malignant neoplasm of rectum
Change in bowel habit	19EA.00	Change in bowel habit
	19EA.11	Altered bowel habit
	19EE.00	Increased frequency of defaecation
	J521200	IBS characterised by alternating bowel habit
	R078.00	[D]Change in bowel habit
Constipation	19C..00	Constipation
	19C..11	Constipation symptom
	19C..12	Costive symptom
	19C2.00	Constipated
	19CZ.00	Constipation NOS
	E264500	Psychogenic constipation
	J520.00	Constipation - functional
	J520000	Acute constipation
	J520100	Chronic constipation with overflow
	J520200	Chronic constipation without overflow
	J520400	Chronic constipation
	J520y00	Other specified constipation
	J520z00	Constipation NOS
	J521100	Irritable bowel syndrome characterised by constipation
	J52y100	Difficulty in ability defaecat
Diarrhoea	19E3.00	Incontinent of faeces
	19E3.11	Incontinent of faeces symptom
	19EE.00	Increased frequency of defaecation
	19EF.00	Urgent desire for stool
	19F..00	Diarrhoea symptoms
	19F..11	Diarrhoea

For peer review only

	19F..12	Loose stools
	19F2.00	Diarrhoea
	19F3.00	Spurious (overflow) diarrhoea
	19F5.00	Time since last episode of diarrhoea
	19FZ.00	Diarrhoea symptom NOS
	19FZ.11	Diarrhoea & vomiting, symptom
	19G..00	Diarrhoea and vomiting
	E264300	Psychogenic diarrhoea
	E264311	Spurious diarrhoea
	Eu45317	[X]Psychogenic diarrhoea
	J4...13	Noninfective diarrhoea
	J43z.11	Chronic diarrhoea
	J4z..11	Presumed noninfectious diarrhoea
	J4zz.11	Diarrhoea - presumed non-infectious
	J521000	Irritable bowel syndrome with diarrhoea
	J525.00	Functional diarrhoea
	J528.00	Intestinal hurry
	R077100	[D] Stools loose
Bloating	19A..00	Abdominal distension symptom
	19A2.00	Abdomen feels bloated
	19A3.00	Abdomen feels distended
	19A4.00	Abdomen feels swollen
	19AZ.00	Abd. distension symptom NOS
	19B..00	Flatulence/wind
	19B..12	Bloating symptom
	19B..14	Flatulence symptom
	19B..15	Wind symptom
	19B2.00	Excessive flatulence
	19B5.00	Excessive flatus
	19BZ.00	Wind NOS
	Eu45320	[X]Psychogenic flatulence
	R073.00	[D]Flatulence, eructation and gas pain
	R073000	[D]Flatulence
	R073200	[D]Gas pain (abdominal)
	R073300	[D]Abdominal distension, gaseous
	R073400	[D]Bloating
	R073500	[D]Tympanites (abdominal)
	R073z00	[D]Flatulence, eructation and gas pain NOS
	R073z11	[D]Wind
Abdominal pain	196..11	Abdominal pain type
	1962.00	Colicky abdominal pain
	1963.00	Non-colicky abdominal pain

For peer review only

	1967.00	Abdominal migraine - symptom
	1968.00	Abdominal discomfort
	1969.00	Abdominal pain
	1969000	Abdominal wall pain
	197..11	Flank pain
	197..12	Iliac fossa pain
	197..13	Site of abdominal pain
	197..14	Subcostal pain
	1971.00	Central abdominal pain
	1972.00	Epigastric pain
	1973.00	Left subcostal pain
	1974.00	Right subcostal pain
	1975.00	Left flank pain
	1976.00	Right flank pain
	1977.00	Right iliac fossa pain
	1978.00	Left iliac fossa pain
	1979.00	Suprapubic pain
	197A.00	Generalised abdominal pain
	197A.11	General abdominal pain-symptom
	197B.00	Upper abdominal pain
	197C.00	Lower abdominal pain
	197D.00	Right upper quadrant pain
	25C..00	O/E - abdo. pain on palpation
	25C..11	O/E - epigastric pain on palp.
	25C..12	O/E - iliac pain on palpation
	25C..14	O/E - umbilical pain on palp.
	25C..15	O/E - abdomen tender
	25C2.00	O/E - abd.pain-R.hypochondrium
	25C3.00	O/E - abd. pain - epigastrium
	25C4.00	O/E - abd.pain-L.hypochondrium
	25C5.00	O/E - abd. pain - R.lumbar
	25C6.00	O/E - abd. pain - umbilical
	25C7.00	O/E - abd. pain - L.lumbar
	25C8.00	O/E - abd. pain - R.iliac
	25C9.00	O/E - abd. pain - hypogastrium
	25CA.00	O/E - abd. pain - L.iliac
	25CZ.00	O/E -abd.pain on palpation NOS
	F262200	Abdominal migraine
	R073200	[D]Gas pain (abdominal)
	R090.00	[D]Abdominal pain
	R090000	[D]Abdominal tenderness
	R090100	[D]Abdominal colic
	R090400	[D]Abdominal cramps
	R090500	[D]Epigastric pain

For peer review only

	R090600	[D]Umbilical pain
	R090700	[D]Hypochondrial pain
	R090800	[D]Suprapubic pain
	R090900	[D]Pain in right iliac fossa
	R090A00	[D]Pain in left iliac fossa
	R090C00	[D]Loin pain
	R090D00	[D]Abdominal migraine
	R090E00	[D]Recurrent acute abdominal pain
	R090F00	[D]Acute abdomen
	R090G11	[D] Pelvic pain
	R090H00	[D]Upper abdominal pain
	R090J00	[D]Right upper quadrant pain
	R090K00	[D]Left upper quadrant pain
	R090L00	[D]Left lower quadrant pain
	R090M00	[D]Right lower quadrant pain
	R090N00	[D]Nonspecific abdominal pain
	R090P00	[D]Functional abdominal pain syndrome
	R090y00	[D]Other specified abdominal pain
	R090z00	[D]Abdominal pain NOS
	Ryu1000	[X]Pain localized to other parts of lower abdomen
	Ryu1100	[X]Other and unspecified abdominal pain
FC test	47J..00	Faecal calprotectin content
	47J0.00	Faecal calprotectin test indeterminate
	47J1.00	Faecal calprotectin test invalid
	47J2.00	Faecal calprotectin test positive
	47J3.00	Faecal calprotectin test negative
	4Q3J.00	Calprotectin level

For peer review only

Supplementary Table 2 Patient characteristics with FC tests included and excluded from the test accuracy study

	Included FC tests (N=5970)	Excluded FC tests (N=9370)	p-value
Age (mean (SD)) at FC test date, range	44.07 (16.23), 18-101	43.42 (16.54), 18-97	0.01594
Sex (female (%))	3828 (64.1)	5963 (63.6)	0.5452
BMI (mean (SD))	26.86 (6.25)	26.93 (6.22)	0.5285
Not reported n (%)	656 (10.99)	1061 (11.32)	0.521
Ethnicity n (%)			
White	3441 (57.64)	5507 (58.77)	0.1647
Asian	93 (1.56)	249 (2.66)	<0.0001
Black	63 (1.06)	135 (1.44)	0.0392
Mixed	24 (0.40)	64 (0.68)	0.0246
Other	33 (0.55)	55 (0.59)	0.7844
Not reported	2316 (38.79)	3360 (35.86)	0.0002
Townsend score n (%)			
1 (least deprived)	988 (16.55)	1936 (20.66)	<0.0001
2	1101 (18.44)	1691 (18.05)	0.5362
3	1140 (19.10)	1720 (18.36)	0.2518
4	1135 (19.01)	1666 (17.78)	0.0542
5 (most deprived)	1062 (17.79)	1149 (12.26)	<0.0001
Missing	544 (9.11)	1208 (12.89)	<0.0001
Country (n) (%)			
England	3748 (62.78)	7073 (75.49)	<0.0001
Northern Ireland	16 (0.27)	566 (6.04)	<0.0001
Scotland	1916 (32.09)	1007 (10.75)	<0.0001
Wales	290 (4.86)	724 (7.73)	<0.0001
CRC ever recorded n (%)	31 (0.52)	45 (0.48)	0.7373
IBS ever recorded n (%)	1897 (31.78)	2649 (28.27)	<0.0001
IBD ever recorded n (%)	208 (3.48)	208 (2.22)	<0.0001
IBD + IBS recorded n (%)	78 (1.31)	63 (0.67)	<0.0001
IBD + CRC recorded n (%)	2 (0.03)	0 (0)	0.0764
No diagnosis recorded n (%)	3754 (62.88)	6405 (68.36)	<0.0001

There were regional differences, with more FC tests excluded from Wales and Northern Ireland and fewer excluded from Scotland and England. There were also more excluded from the least deprived group according to Townsend Score, and fewer excluded from the most deprived group.

Supplementary Table 3 Test accuracy measures of FC testing at threshold of 100µg/g compared to 50µg/g

Threshold	N	TP	FP	FN	TN	Sensitivity, % (95% CI)	Specificity, % (95% CI)	PPV, % (95% CI)	NPV, % (95% CI)
50µg/g	5970	195	2219	15	3541	92.9 (88.6 to 95.6)	61.5 (60.2 to 62.7)	8.1 (7.1 to 9.2)	99.6 (99.3 to 99.7)
100µg/g	5970	181	1344	29	4416	86.2 (80.9 to 90.2)	76.7 (75.6 to 77.7)	11.9 (10.3 to 13.6)	99.3 (99.1 to 99.5)
250µg/g	5970	130	563	80	5197	61.9 (55.2 to 68.2)	90.2 (89.4 to 91)	18.8 (16 to 21.8)	98.5 (98.1 to 98.8)

N sample size, TP true positives, FP false positives, FN false negatives, TN true negatives, PPV positive predictive value, NPV negative predictive value, CI confidence interval

For peer review only

Section & Topic	No	Item	Reported on page #
**TITLE OR ABSTRACT**			
	**1**	Identification as a study of diagnostic accuracy using at least one measure of accuracy (such as sensitivity, specificity, predictive values, or AUC)	1, 2
**ABSTRACT**			
	**2**	Structured summary of study design, methods, results, and conclusions (for specific guidance, see STARD for Abstracts)	2
**INTRODUCTION**			
	**3**	Scientific and clinical background, including the intended use and clinical role of the index test	4
	**4**	Study objectives and hypotheses	4
**METHODS**			
Study design	**5**	Whether data collection was planned before the index test and reference standard were performed (prospective study) or after (retrospective study)	5
Participants	**6**	Eligibility criteria	5
	**7**	On what basis potentially eligible participants were identified (such as symptoms, results from previous tests, inclusion in registry)	5
	**8**	Where and when potentially eligible participants were identified (setting, location and dates)	5
	**9**	Whether participants formed a consecutive, random or convenience series	NA all eligible patients in database were included
Test methods	**10a**	Index test, in sufficient detail to allow replication	5
	**10b**	Reference standard, in sufficient detail to allow replication	5 and Supplementary table 1
	**11**	Rationale for choosing the reference standard (if alternatives exist)	NA
	**12a**	Definition of and rationale for test positivity cut-offs or result categories of the index test, distinguishing pre-specified from exploratory	5
	**12b**	Definition of and rationale for test positivity cut-offs or result categories of the reference standard, distinguishing pre-specified from exploratory	5 and 6
	**13a**	Whether clinical information and reference standard results were available to the performers/readers of the index test	NA routine data
	**13b**	Whether clinical information and index test results were available to the assessors of the reference standard	NA routine data
Analysis	**14**	Methods for estimating or comparing measures of diagnostic accuracy	5 and 6
	**15**	How indeterminate index test or reference standard results were handled	Figure 1
	**16**	How missing data on the index test and reference standard were handled	5, Figure 1
	**17**	Any analyses of variability in diagnostic accuracy, distinguishing pre-specified from exploratory	6
	**18**	Intended sample size and how it was determined	NA all eligible patients from database included
**RESULTS**			
Participants	**19**	Flow of participants, using a diagram	Figure 1
	**20**	Baseline demographic and clinical characteristics of participants	Supplementary table 2
	**21a**	Distribution of severity of disease in those with the target condition	NA
	**21b**	Distribution of alternative diagnoses in those without the target condition	NA
	**22**	Time interval and any clinical interventions between index test and reference standard	NA
Test results	**23**	Cross tabulation of the index test results (or their distribution) by the results of the reference standard	Tables 1, 2, 3 and supplementary table 3
	**24**	Estimates of diagnostic accuracy and their precision (such as 95% confidence intervals)	Tables 1, 2, 3 and supplementary table 3
	**25**	Any adverse events from performing the index test or the reference standard	NA

For peer review only

**DISCUSSION**			
	**26**	Study limitations, including sources of potential bias, statistical uncertainty, and generalisability	12-13
	**27**	Implications for practice, including the intended use and clinical role of the index test	14-15
**OTHER INFORMATION**			
	**28**	Registration number and name of registry	NA
	**29**	Where the full study protocol can be accessed	5
	**30**	Sources of funding and other support; role of funders	20

For peer review only

STARD 2015

AIM

STARD stands for "Standards for Reporting Diagnostic accuracy studies". This list of items was developed to contribute to the completeness and transparency of reporting of diagnostic accuracy studies. Authors can use the list to write informative study reports. Editors and peer-reviewers can use it to evaluate whether the information has been included in manuscripts submitted for publication.

EXPLANATION

A **diagnostic accuracy study** evaluates the ability of one or more medical tests to correctly classify study participants as having a **target condition.** This can be a disease, a disease stage, response or benefit from therapy, or an event or condition in the future. A medical test can be an imaging procedure, a laboratory test, elements from history and physical examination, a combination of these, or any other method for collecting information about the current health status of a patient.

The test whose accuracy is evaluated is called **index test.** A study can evaluate the accuracy of one or more index tests. Evaluating the ability of a medical test to correctly classify patients is typically done by comparing the distribution of the index test results with those of the **reference standard**. The reference standard is the best available method for establishing the presence or absence of the target condition. An accuracy study can rely on one or more reference standards.

If test results are categorized as either positive or negative, the cross tabulation of the index test results against those of the reference standard can be used to estimate the **sensitivity** of the index test (the proportion of participants *with* the target condition who have a positive index test), and its **specificity** (the proportion *without* the target condition who have a negative index test). From this cross tabulation (sometimes referred to as the contingency or "2x2" table), several other accuracy statistics can be estimated, such as the positive and negative **predictive values** of the test. Confidence intervals around estimates of accuracy can then be calculated to quantify the statistical **precision** of the measurements.

If the index test results can take more than two values, categorization of test results as positive or negative requires a **test positivity cut-off**. When multiple such cut-offs can be defined, authors can report a receiver operating characteristic (ROC) curve which graphically represents the combination of sensitivity and specificity for each possible test positivity cut-off. The **area under the ROC curve** informs in a single numerical value about the overall diagnostic accuracy of the index test.

The **intended use** of a medical test can be diagnosis, screening, staging, monitoring, surveillance, prediction or prognosis. The **clinical role** of a test explains its position relative to existing tests in the clinical pathway. A replacement test, for example, replaces an existing test. A triage test is used before an existing test; an add-on test is used after an existing test.

Besides diagnostic accuracy, several other outcomes and statistics may be relevant in the evaluation of medical tests. Medical tests can also be used to classify patients for purposes other than diagnosis, such as staging or prognosis. The STARD list was not explicitly developed for these other outcomes, statistics, and study types, although most STARD items would still apply.

DEVELOPMENT

This STARD list was released in 2015. The 30 items were identified by an international expert group of methodologists, researchers, and editors. The guiding principle in the development of STARD was to select items that, when reported, would help readers to judge the potential for bias in the study, to appraise the applicability of the study findings and the validity of conclusions and recommendations. The list represents an update of the first version, which was published in 2003.

More information can be found on http://www.equator-network.org/reporting-guidelines/stard.

For peer review only

CC BY SA

The RECORD statement – checklist of items, extended from the STROBE statement, that should be reported in observational studies using routinely collected health data.

	Item No.	STROBE items	Location in manuscript where items are reported	RECORD items	Location in manuscript where items are reported
**Title and abstract**					
	1	(a) Indicate the study's design with a commonly used term in the title or the abstract (b) Provide in the abstract an informative and balanced summary of what was done and what was found	p1-2	RECORD 1.1: The type of data used should be specified in the title or abstract. When possible, the name of the databases used should be included.<br><br>RECORD 1.2: If applicable, the geographic region and timeframe within which the study took place should be reported in the title or abstract.<br><br>RECORD 1.3: If linkage between databases was conducted for the study, this should be clearly stated in the title or abstract.	P1<br><br>p2<br><br>NA
**Introduction**					
Background rationale	2	Explain the scientific background and rationale for the investigation being reported	p4		
Objectives	3	State specific objectives, including any prespecified hypotheses	p4		
**Methods**					
Study Design	4	Present key elements of study design early in the paper	p1, 5		
Setting	5	Describe the setting, locations, and relevant dates, including periods of recruitment, exposure, follow-up, and data collection	P5		

For peer review only

Participants	6	*(a) Cohort study* - Give the eligibility criteria, and the sources and methods of selection of participants. Describe methods of follow-up<br>*Case-control study* - Give the eligibility criteria, and the sources and methods of case ascertainment and control selection. Give the rationale for the choice of cases and controls<br>*Cross-sectional study* - Give the eligibility criteria, and the sources and methods of selection of participants<br><br>*(b) Cohort study* - For matched studies, give matching criteria and number of exposed and unexposed<br>*Case-control study* - For matched studies, give matching criteria and the number of controls per case	p5-6<br><br>NA	RECORD 6.1: The methods of study population selection (such as codes or algorithms used to identify subjects) should be listed in detail. If this is not possible, an explanation should be provided.<br><br>RECORD 6.2: Any validation studies of the codes or algorithms used to select the population should be referenced. If validation was conducted for this study and not published elsewhere, detailed methods and results should be provided.<br><br>RECORD 6.3: If the study involved linkage of databases, consider use of a flow diagram or other graphical display to demonstrate the data linkage process, including the number of individuals with linked data at each stage.	Supplementary Table 1<br><br>p5<br><br>NA
Variables	7	Clearly define all outcomes, exposures, predictors, potential confounders, and effect modifiers. Give diagnostic criteria, if applicable.	p5-6	RECORD 7.1: A complete list of codes and algorithms used to classify exposures, outcomes, confounders, and effect modifiers should be provided. If these cannot be reported, an explanation should be provided.	Supplementary Table 1
Data sources/ measurement	8	For each variable of interest, give sources of data and details of methods of assessment (measurement).<br>Describe comparability of assessment methods if there is more than one group	p5		

For peer review only

Bias	9	Describe any efforts to address potential sources of bias	p6		
Study size	10	Explain how the study size was arrived at	NA		
Quantitative variables	11	Explain how quantitative variables were handled in the analyses. If applicable, describe which groupings were chosen, and why	p12		
Statistical methods	12	(a) Describe all statistical methods, including those used to control for confounding<br>(b) Describe any methods used to examine subgroups and interactions<br>(c) Explain how missing data were addressed<br>(d) *Cohort study* - If applicable, explain how loss to follow-up was addressed<br>*Case-control study* - If applicable, explain how matching of cases and controls was addressed<br>*Cross-sectional study* - If applicable, describe analytical methods taking account of sampling strategy<br>(e) Describe any sensitivity analyses	p6		
Data access and cleaning methods		..		RECORD 12.1: Authors should describe the extent to which the investigators had access to the database population used to create the study population.	p20

For peer review only

				RECORD 12.2: Authors should provide information on the data cleaning methods used in the study.	Figure 1
Linkage		..		RECORD 12.3: State whether the study included person-level, institutional-level, or other data linkage across two or more databases. The methods of linkage and methods of linkage quality evaluation should be provided.	NA
**Results**					
Participants	13	(a) Report the numbers of individuals at each stage of the study (*e.g.*, numbers potentially eligible, examined for eligibility, confirmed eligible, included in the study, completing follow-up, and analysed)<br>(b) Give reasons for non-participation at each stage.<br>(c) Consider use of a flow diagram	Figure 1	RECORD 13.1: Describe in detail the selection of the persons included in the study (*i.e.,* study population selection) including filtering based on data quality, data availability and linkage. The selection of included persons can be described in the text and/or by means of the study flow diagram.	Figure 1
Descriptive data	14	(a) Give characteristics of study participants (*e.g.*, demographic, clinical, social) and information on exposures and potential confounders<br>(b) Indicate the number of participants with missing data for each variable of interest<br>(c) *Cohort study* - summarise follow-up time (*e.g.*, average and total amount)	Supplementary Table 2		
Outcome data	15	*Cohort study* - Report numbers of outcome events or summary measures over time<br>*Case-control study* - Report numbers in each exposure	Tables 1 and 2		

For peer review only

		category, or summary measures of exposure<br>*Cross-sectional study* - Report numbers of outcome events or summary measures			
Main results	16	(a) Give unadjusted estimates and, if applicable, confounder-adjusted estimates and their precision (e.g., 95% confidence interval). Make clear which confounders were adjusted for and why they were included<br>(b) Report category boundaries when continuous variables were categorized<br>(c) If relevant, consider translating estimates of relative risk into absolute risk for a meaningful time period	p7-9		
Other analyses	17	Report other analyses done—e.g., analyses of subgroups and interactions, and sensitivity analyses	p9-10		
**Discussion**					
Key results	18	Summarise key results with reference to study objectives	p12		
Limitations	19	Discuss limitations of the study, taking into account sources of potential bias or imprecision. Discuss both direction and magnitude of any potential bias	p12-13	RECORD 19.1: Discuss the implications of using data that were not created or collected to answer the specific research question(s). Include discussion of misclassification bias, unmeasured confounding, missing data, and changing eligibility over time, as they pertain to the study being reported.	p12-13
Interpretation	20	Give a cautious overall interpretation of results considering objectives,	p14-15		

		limitations, multiplicity of analyses, results from similar studies, and other relevant evidence			
Generalisability	21	Discuss the generalisability (external validity) of the study results	p13-14		
**Other Information**					
Funding	22	Give the source of funding and the role of the funders for the present study and, if applicable, for the original study on which the present article is based	p20		
Accessibility of protocol, raw data, and programming code		..		RECORD 22.1: Authors should provide information on how to access any supplemental information such as the study protocol, raw data, or programming code.	p20

*Reference: Benchimol EI, Smeeth L, Guttmann A, Harron K, Moher D, Petersen I, Sørensen HT, von Elm E, Langan SM, the RECORD Working Committee. The REporting of studies Conducted using Observational Routinely-collected health Data (RECORD) Statement. *PLoS Medicine* 2015; in press.

*Checklist is protected under Creative Commons Attribution (CC BY) license.

For peer review only

BMJ Open

Test Accuracy of Faecal Calprotectin for Inflammatory Bowel Disease in UK Primary Care: Retrospective Cohort Study of the IMRD-UK data

Journal:	*BMJ Open*
Manuscript ID	bmjopen-2020-044177.R1
Article Type:	Original research
Date Submitted by the Author:	12-Nov-2020
Complete List of Authors:	Freeman, Karoline; University of Warwick, Warwick Medical School; University of Warwick<br>Taylor-Phillips, Sian; University of Warwick, Warwick Medical School<br>Willis, Brian; University of Birmingham, Institute of Applied Health Research<br>Ryan, Ronan; University of Birmingham, Institute of Applied Health Research<br>Clarke, Aileen; University of Warwick, Division of Health Sciences
**Primary Subject Heading**:	General practice / Family practice
Secondary Subject Heading:	Gastroenterology and hepatology
Keywords:	Inflammatory bowel disease < GASTROENTEROLOGY, PRIMARY CARE, GASTROENTEROLOGY

SCHOLARONE™
Manuscripts

I, the Submitting Author has the right to grant and does grant on behalf of all authors of the Work (as defined in the below author licence), an exclusive licence and/or a non-exclusive licence for contributions from authors who are: i) UK Crown employees; ii) where BMJ has agreed a CC-BY licence shall apply, and/or iii) in accordance with the terms applicable for US Federal Government officers or employees acting as part of their official duties; on a worldwide, perpetual, irrevocable, royalty-free basis to BMJ Publishing Group Ltd ("BMJ") its licensees and where the relevant Journal is co-owned by BMJ to the co-owners of the Journal, to publish the Work in this journal and any other BMJ products and to exploit all rights, as set out in our licence.

The Submitting Author accepts and understands that any supply made under these terms is made by BMJ to the Submitting Author unless you are acting as an employee on behalf of your employer or a postgraduate student of an affiliated institution which is paying any applicable article publishing charge ("APC") for Open Access articles. Where the Submitting Author wishes to make the Work available on an Open Access basis (and intends to pay the relevant APC), the terms of reuse of such Open Access shall be governed by a Creative Commons licence – details of these licences and which Creative Commons licence will apply to this Work are set out in our licence referred to above.

Other than as permitted in any relevant BMJ Author's Self Archiving Policies, I confirm this Work has not been accepted for publication elsewhere, is not being considered for publication elsewhere and does not duplicate material already published. I confirm all authors consent to publication of this Work and authorise the granting of this licence.

For peer review only

Test Accuracy of Faecal Calprotectin for Inflammatory Bowel Disease in UK Primary Care: Retrospective Cohort Study of the IMRD-UK data

Karoline Freeman (0000-0002-9963-2918), Sian Taylor-Phillips, Brian H Willis, Ronan Ryan, Aileen Clarke

Karoline Freeman NIHR Doctoral Research Fellow, Division of Health Sciences, University of Warwick, Coventry, CV4 7AL, UK

Sian Taylor-Phillips Professor of Screening and Test Evaluation, Division of Health Sciences, University of Warwick, Coventry, CV4 7AL, UK

Brian H Willis MRC Clinician Scientist, Institute of Applied Health Research, University of Birmingham, Birmingham, B15 2TT, UK

Ronan Ryan Research Fellow, Institute of Applied Health Research, University of Birmingham, Birmingham, B15 2TT, UK

Aileen Clarke Professor of Public Health & Health Services Research, Division of Health Sciences, University of Warwick, Coventry, CV4 7AL, UK

Correspondence to:
Karoline Freeman: k.freeman@warwick.ac.uk

Words: 4094

For peer review only - http://bmjopen.bmj.com/site/about/guidelines.xhtml

ABSTRACT

Objective: To estimate the test accuracy of faecal calprotectin (FC) for inflammatory bowel disease (IBD) in the primary care setting using routine electronic health records.

Design: Retrospective cohort test accuracy study

Setting: UK primary care

Participants: 5970 patients (≥18 years) without a previous IBD diagnosis and with a first FC test between 1 January 2006 and 31 December 2016. We excluded multiple tests and tests without numeric results in units of μg/g.

Intervention: FC testing for the diagnosis of IBD. Disease status was confirmed by a recorded diagnostic code and/or a drug code of an IBD specific medication at three time points after the FC test date.

Main outcome measures: Sensitivity, specificity, positive and negative predictive values for the differential of IBD versus non-IBD and IBD versus irritable bowel syndrome (IBS) at the 50μg/g and 100μg/g thresholds.

Results: 5970 patients met the inclusion criteria and had at least 6 months of follow-up data after FC testing. 1897 had an IBS diagnosis, 208 had an IBD diagnosis, 31 had a colorectal cancer diagnosis, 80 had more than one diagnosis and 3754 had no subsequent diagnosis. Sensitivity, specificity, positive and negative predictive values were 92.9% (88.6% to 95.6%), 61.5% (60.2% to 62.7%), 8.1% (7.1% to 9.2%) and 99.6% (99.3% to 99.7%), respectively at the threshold of 50μg/g. Raising the threshold to 100μg/g missed less than 7% additional IBD cases. Longer follow-up had no effect on test accuracy. Overall, uncertainty was greater for specificity than sensitivity. General practitioners' referral decisions did not follow the anticipated clinical pathways in national guidance.

Conclusions: GPs can be confident in excluding IBD on the basis of a negative FC test in a population with low pre-test risk but should interpret a positive test with caution. The applicability of national guidance to general practice needs to be improved.

For peer review only - http://bmjopen.bmj.com/site/about/guidelines.xhtml

Strengths and limitations of this study

- The sample size in our study is likely to be larger than the usual cross-sectional studies used to evaluate a test, which is useful for IBD which has a low prevalence.
- We applied strict eligibility criteria to ensure the reliability of reported test accuracy measures and explored the effects of different follow-up times on the definition of IBD disease.
- We used a very comprehensive and sensitive list of Read codes and drug codes (48 codes) for the identification of IBD.
- The exclusion of close to half of the FC tests without a numerical test result raises concerns over the generalisability of the findings, however, findings are in line with published estimates.
- A coded IBD diagnosis or prescription is a proxy reference standard for a composite of different reference tests used in clinical practice reflecting a pragmatic approach to test accuracy.

For peer review only

For peer review only - http://bmjopen.bmj.com/site/about/guidelines.xhtml

INTRODUCTION

Inflammatory bowel disease is a chronic, progressive disorder caused by inflammation of the gastrointestinal tract. Prevalence of IBD is rising worldwide with highest reported prevalence values in North America (568 per 100,000 persons) and Europe (827 per 100,000 persons).[1] UK prevalence estimates were 970/100,000 in 2017.[2] It often presents with non-specific symptoms such as abdominal pain and diarrhoea making it difficult to differentiate from irritable bowel syndrome (IBS), which is far more prevalent in the primary care setting. This also affects the numbers and composition of referrals to secondary care - with a higher proportion of patients being diagnosed in secondary care with IBS than is desirable. It is against this background that the non-invasive faecal calprotectin test has been introduced as a test for IBD in primary care in the UK. Faecal calprotectin (FC) is a small calcium-binding protein of the S100 family which is predominantly derived from neutrophils during an immune response. It is associated with inflammation and can be quantified in stool samples.[3] Therefore, it is more specific than serum inflammatory markers in the detection of gastrointestinal inflammation.[3]

The FC test is recommended by gastroenterological societies across the globe for its usefulness in the diagnosis of IBD.[4-7] However, there is no clear guidance on which settings it is considered appropriate. The National Institute for Health and Care Excellence (NICE) recommends the use of FC testing in UK primary care for the differential diagnosis of IBD and IBS when a referral is being considered and colorectal cancer is not suspected.[8] A study of routine data from an American health plans database of FC testing for chronic gastrointestinal symptoms in primary care suggests that it is used in routine general practice in at least one other country.[9] The NICE recommendations were based on an evaluation of secondary care test accuracy studies and assumptions that referral decisions would follow FC test results. This resulted in a slow uptake and inconsistent use of FC testing in primary care.[10] While primary care studies exist, we still lack compelling evidence on the test accuracy of FC testing in primary care for the detection of IBD. This is because the primary care evidence is based on small, heterogeneous studies that vary in clinical questions, FC thresholds and definitions of disease.[11] They report a range of sensitivities and specificities with wide confidence intervals and leave uncertainty over which threshold (50 or 100µg/g) should be adopted in primary care. To determine the utility of FC testing in primary care, a pragmatic study is required that both captures the differential diagnosis between IBD and IBS and has a patient population that is representative of primary care.

For peer review only - http://bmjopen.bmj.com/site/about/guidelines.xhtml

Thus, our primary aim was to determine the diagnostic value of FC for IBD in primary care by estimating the sensitivity, specificity and predictive values for two thresholds using routine UK primary care data from the IQVIA Medical Research Database (IMRD)-UK.

METHODS

Data source

We undertook a test accuracy study of FC testing for IBD using data from the IMRD-UK (formerly known as The Health Improvement Network (THIN)). IMRD-UK is a UK longitudinal primary care database of over 14 million patients from more than 670 GP practices covering about 6% of the UK population in 2015.[12] It has been found to be broadly representative of the UK population.[13] Patients' medical diagnoses, symptoms, laboratory tests, referrals and prescriptions are available as clinical codes (e.g. Read codes). The study was approved by the Independent Scientific Advisory Committee (protocol number 17THIN089).

Study design and population

This retrospective cohort study included patients (≥18 years) who had no previous IBD diagnosis and who had their first FC test between 1 January 2006 and 31 December 2016. Eligible patients had also contributed at least 12 months of data to IMRD-UK before their study start date. FC tests were identified using all six Read codes available to GPs to record an FC test.

Classification of FC test results

FC tests with a numeric result >50μg/g and >100μg/g were considered positive in two separate analyses. We included a post-hoc analysis at the threshold of 250μg/g in light of the latest recommendations of a pathway using two cut-offs.[10, 14] FC tests without results, with invalid results, qualitative results only, missing units of measurement, or units of measurement other than μg/g were excluded from the analysis.

Definition of disease

IBD was defined as a record of a Read code for ulcerative colitis, Crohn's disease, indeterminate colitis or microscopic colitis. Read code lists were adapted from those used in previous literature.[15-17] This was supplemented with the record of an IBD specific prescription to increase the sensitivity of the algorithm. IBS and colorectal cancer (CRC) were defined by

For peer review only - http://bmjopen.bmj.com/site/about/guidelines.xhtml

any clinical code that suggested a definitive diagnosis. Abdominal symptoms were not considered sufficiently specific for an IBS diagnosis. We excluded benign and precancerous stages of CRC. In three different analyses a diagnosis was considered when it was recorded within 6 months, 12 months or 24 months of the FC test date to explore the impact of late diagnoses in patients with sufficient follow-up after FC testing. We considered the following symptoms to assess eligibility for testing: diarrhoea, abdominal pain, constipation, bloating and change in bowel habit. See Supplementary Table 1 for final code lists of these variables.

Definition of referral

Our definition of IBD was based on the assumption that IBD diagnoses were only recorded following confirmatory testing in secondary care. We explored this assumption by investigating FC testing in the referred population only. We considered a referral to any speciality that was made within six weeks of the index FC test to be a referral in response to FC testing.

Statistical analysis

Data management and analysis were undertaken in R version 3.6.1 (Vienna, Austria).[18] The main outcomes of the analysis were sensitivity, specificity, and positive and negative predictive values. These were calculated in two ways: 1) Patients with a recorded IBD diagnosis were classed as disease positive and those without an IBD diagnosis (IBS, CRC, alternative diagnosis, no diagnosis) were classed as disease negative. (2) Patients with an IBD diagnosis were classed as disease positive and only those with an IBS diagnosis were classed as disease negative. Confidence intervals for proportions were computed using the Wilson method.[19] We used the Koopman method to compute confidence intervals for likelihood ratios.[20]

We used receiver operating characteristic (ROC) curves to display the accuracy of the FC test at test positive thresholds between 33 and 300µg/g. This allowed the inclusion of results recorded as >300µg/g and <33µg/g. ROC curves for different follow-up times were compared using Venkatraman's method for two unpaired ROC curves in the absence of a feasible paired comparison because data were of different sizes.[21]

We computed proportions of patients referred with a true positive (TP), false positive (FP), false negative (FN) and true negative (TN) FC test result in order to investigate the effect the FC test results have on patient management. If the test results had no impact on referral, the proportions across groups were expected to be similar. If GPs act on the test result (refer

For peer review only - http://bmjopen.bmj.com/site/about/guidelines.xhtml

positives and manage negatives), the proportion of patients referred with TP and FP results was expected to be 1) close to one; 2) equal; and 3) greater than those for FN and TN results (both close to 0). The proportions of referred patients were compared using the two proportions z-test.

Sensitivity analyses were conducted in order to investigate the effects different assumptions have on the test accuracy estimates. Specifically, we investigated: including all units of measurements; including qualitative results in addition to quantitative results; defining IBD by Read code only; and confining analyses to only those patients referred to secondary care. We also explored the effects of excluding patients not eligible for the FC testing as according to national guidance (patients with an age >50 years or symptoms for <6 weeks).

Patient involvement

A patient advisory group was actively involved in the funding application for the project. The group provided a patient perspective in the design of the study and the interpretation of study findings.

RESULTS

17,466 first time FC tests in a total of 6,965,853 adult primary care patients were recorded in the study period, and 5,970 were included in the analysis (Figure 1). 1,987 (32%) had an IBS diagnosis, 208 (3.5%) an IBD diagnosis, 31 (0.5%) a colorectal cancer (CRC) diagnosis, 78 (1%) had an IBD and an IBS diagnosis, 2 had an IBD and a CRC diagnosis and 3,754 (63%) had no diagnosis recorded at any time after the FC test (see online Supplementary Table 2). 1,434/5970 (24%) patients had an IBS diagnosis recorded at the time of FC testing. The proportion of patients with an IBD or IBS diagnosis were slightly higher in the included than the excluded group (see online Supplementary Table 2).

++ Figure 1 ++

Test accuracy of FC testing for IBD

In 5,970 patients with at least 6 months follow-up available after the FC test date (Figure 1), the sensitivity of the FC test in detecting IBD at a threshold of 50µg/g was 92.9% (95% CI 88.6% to 95.6%). Specificity was 61.5% (95% CI 60.2% to 62.7%). The positive predictive value was low at 8.1% (95% CI 7.1% to 9.2%) while the negative predictive value was high (99.6% [95% CI 99.3% to 99.7%]) at an IBD prevalence of 3.5% (Table 1). The positive likelihood ratio was 2.41 (95%CI 2.28 to 2.52) which suggests that FC testing only slightly

For peer review only - http://bmjopen.bmj.com/site/about/guidelines.xhtml

increases the probability of IBD disease. The negative likelihood ratio was 0.12 (95% CI 0.07 to 0.19) which suggests strong evidence to rule out IBD. The inclusion of IBD cases that were recorded up to 12 (analysis of 4793 FC tested patients) and 24 months (2662 patients) following an FC test had no significant effect on the measures of test accuracy (Table 1). Small changes observed in the point estimates may be due to the different population size and the different make-up of the study population. 87% (2,096/2,414) of patients with a positive FC test result had no recorded diagnosis of IBD, IBS or CRC six months after the FC test date. Of these 501 had a previous diagnosis of IBS or CRC. At the end of the study period there were still 1,470 (61%) patients with a positive test but without a diagnosis of interest. These patients could have either have had a missed diagnosis, no diagnosis, or an alternative diagnosis, which was not included in our study. For patients with a negative FC test 92% (3,277/3,556) had none of the three diagnoses recorded at six months and 64% (2,284/3,556) had none of the three diagnoses at any time after FC testing.

Table 1 Test accuracy measures of FC testing at threshold of 50µg/g

**Time***	**N**	**TP**	**FP**	**FN**	**TN**	**Sensitivity, % (95% CI)**	**Specificity, % (95% CI)**	**PPV, % (95% CI)**	**NPV, % (95% CI)**
6 months	5970	195	2219	15	3541	92.9 (88.6 to 95.6)	61.5 (60.2 to 62.7)	8.1 (7.1 to 9.2)	99.6 (99.3 to 99.7)
12 months	4793	198	1790	16	2789	92.5 (88.2 to 95.3)	60.9 (59.5 to 62.3)	10 (8.7 to 11.4)	99.4 (99.1 to 99.6)
24 months	2662	129	1067	11	1455	92.1 (86.5 to 95.6)	57.7 (55.8 to 59.6)	10.8 (9.2 to 12.7)	99.2 (98.7 to 99.6)

*Time following FC testing to consider a record of IBD
N sample size, TP true positives, FP false positives, FN false negatives, TN true negatives, PPV positive predictive value, NPV negative predictive value, CI confidence interval

The ROC curves in Figure 2 describe the sensitivity and false positive rate (1- specificity) for the three different follow-up times at additional thresholds. The 50µg/g threshold prioritises sensitivity over specificity in all three analyses. There was generally good agreement between the three curves (E=0.0016758, p = 0.406 comparing 6 with 24 months follow-up).

++ Figure 2 ++

Increasing the FC thresholds

Figure 3 shows that an increase in the threshold from 50µg/g to 100µg/g affects the specificity more than the sensitivity (online Supplementary Table 3). Increasing the threshold reduces the number of false positives by 875 at the expense of missing an additional 14 cases of IBD in a population of 5,970 tested patients suggesting that the proportion of IBD

For peer review only - http://bmjopen.bmj.com/site/about/guidelines.xhtml

cases missed due to this threshold difference is less than 7% (14/210). IBD cases missed were 31% (65/210) when we changed the threshold to 250µg/g (online Supplementary Table 3).

++ Figure 3 ++

Restricting the population to patients with IBD or IBS

Changing the definition of the non-diseased group from non-IBD to IBS reduced the number of false positives and true negatives. Consequently, the point estimates of specificity and PPV increased by about 10% and 60%, respectively at the 50µg/g threshold (Table 2). However, the dataset was small (576 compared to 5,970) and, thus, increases the potential for sampling error. This may limit the clinical applicability of the findings.

Table 2 Test accuracy measures for FC testing at the threshold of 50µg/g for the detection of IBD recorded within 6 months of FC testing considering two different definitions of the non-diseased group

	**N**	**TP**	**FP**	**FN**	**TN**	**Sensitivity, % (95% CI)**	**Specificity, % (95% CI)**	**PPV, % (95% CI)**	**NPV, % (95% CI)**
IBD vs non-IBD	5970	195	2219	15	3541	92.9 (88.6 to 95.6)	61.5 (60.2 to 62.7)	8.1 (7.1 to 9.2)	99.6 (99.3 to 99.7)
IBD vs IBS	576	195	104	15	262	92.9 (88.6 to 95.6)	71.6 (66.8 to 76)	65.2 (59.7 to 70.4)	94.6 (91.3 to 96.7)

N sample size, TP true positive, FP false positive, FN false negative, TN true negative, CI confidence interval, PPV positive predictive value, NPV negative predictive value, IBD inflammatory bowel disease, IBS irritable bowel syndrome

Sensitivity analyses

Overall, the sensitivity analyses revealed greater uncertainty in the estimate of specificity than sensitivity (Table 3). Inclusion of numerical results reported in other units of measurement had no impact on estimates of test accuracy. The majority of qualitative results were positive at the 50µg/g threshold which may indicate preferential recording of positive test results using qualitative codes. In this sensitivity analysis the specificity was lower due to the great number of false positives. Only 569 patients tested had symptoms recorded that met the national guidance eligibility criteria. The recording of symptoms was not associated with the FC test result. Test accuracy measures in this subset did not differ from those estimated using the full dataset. Reclassifying 79 patients as IBD negative who had an IBD diagnosis affirmed by an IBD specific drug code only (Read code negative) had no impact on the test accuracy estimates. Finally, of the 5,970 FC tests, 52% resulted in a

For peer review only - http://bmjopen.bmj.com/site/about/guidelines.xhtml

referral. The specificity of FC testing in this subgroup was lower because of the non-referral of mainly true negatives.

For peer review only

Table 3 Sensitivity analyses compared to primary analysis of FC testing for IBD vs non-IBD at a threshold of 50μg/g with IBD recorded within 6 months

Analysis	N	TP	FP	FN	TN	Sensitivity,% (95% CI)	Specificity, % (95% CI)	PPV, % (95% CI)	NPV, % (95% CI)
Primary analysis	5970	195	2219	15	3541	92.9 (88.6 to 95.6)	61.5 (60.2 to 62.7)	8.1 (7.1 to 9.2)	99.6 (99.3 to 99.7)
FC tests with any unit	6758	220	2481	17	4040	92.8 (88.8 to 95.5)	62 (60.8 to 63.1)	8.1 (7.2 to 9.2)	99.6 (99.3 to 99.7)
Qualitative results included*	7000	261	3177	15	3547	94.6 (91.2 to 96.7)	52.8 (51.6 to 53.9)	7.6 (6.8 to 8.5)	99.6 (99.3 to 99.7)
Eligible according to national guidelines**	569	20	194	1	354	95.2 (77.3 to 99.2)	64.6 (60.5 to 68.5)	9.3 (6.1 to 14)	99.7 (98.4 to 100)
IBD defined by Read code only	5970	148	2266	6	3550	96.1 (91.8 to 98.2)	61 (59.8 to 62.3)	6.1 (5.2 to 7.2)	99.8 (99.6 to 99.9)
Referred only*** (within 6 weeks)	3078	138	1330	11	1599	92.6 (87.3 to 95.8)	54.6 (52.8 to 56.4)	9.4 (8 to 11)	99.3 (98.8 to 99.6)

* with a record of an upper reference range of 50 or above

** Patients <50 years and abdominal symptoms for >6 weeks

*** Assumption that referred patients have high probability of disease verification by colonoscopy

TP true positives, FP false positives, FN false negatives, TN true negatives, PPV positive predictive value, NPV negative predictive value, FC faecal calprotectin

For peer review only

For peer review only - http://bmjopen.bmj.com/site/about/guidelines.xhtml

Impact of test results on referral decisions

Table 4 shows the classification of all included patients and those referred within 6 weeks of their FC test according to their test result and disease status. If GPs referred patients based on positive FC test results, they would have referred 2,414/5,970 (40.4%) included patients. This would have detected 195/210 (92.9%) of IBD cases. However, six weeks after FC testing 3,078/5,970 (51.6%) patients were referred, including 1,610/3,556 (45.3%) FC negative patients and only 149/210 (71.0%) of IBD cases. Therefore, the test result was not the single determining factor for or against a timely referral. Patients with true positive and false negative FC test results were equally likely to be referred for further tests (70.8% vs 73.3%, z=2, p= 0.8371). Both groups were referred more frequently than patients with false positive (59.9%) (z=3, p= 0.0028) or true negative results (45.3%) (z=7, p<0.0001). This may suggest that GPs used alternative cues (in the history, examination or other tests) which raised their suspicion of IBD. Overall, GP assessment plus FC testing may be worse than FC testing alone. Compared to FC testing alone, the number of patients with a true positive result was lower for GP assessment plus testing because GPs did not refer all test positive patients within six weeks. GPs referred some patients with a false negative FC test result on the test, but not enough to make up for not referring patients with true positive results on the test.

Table 4 Proportion of patients referred by test result and disease status

	TP	FP	FN	TN	Total
Classification of included patients in primary analysis based on FC test result n	195	2,219	15	3,541	5,970
Classification of FC tested patients with referral based on FC test result n	138	1,330	11	1,599	3,078
Proportion of patients in primary analysis referred, %	70.8	59.9	73.3	45.3	51.6

DISCUSSION

Study findings

After excluding FC tests with no result or an ambiguous result, the sensitivity of the FC test in detecting IBD at the 50μg/g threshold was high (92.9% [95% CI 88.6% to 95.6%]) but the specificity was modest (61.5% [95% 60.2% to 62.7%]). Moreover, the sensitivity analyses demonstrated greater uncertainty in our estimates of the specificity. The high false positive

For peer review only - http://bmjopen.bmj.com/site/about/guidelines.xhtml

rate may be irrelevant in clinical practice if the group represents alternative inflammatory conditions that warrant further assessment. However, a broader disease category in primary care including other organic conditions in addition to IBD resulted in sensitivity estimates ranging from 64% to 94%.[22-24] As the conditions may follow different pathways, interpretation of a test positive result would still be challenging. Restricting the group without IBD to those with a confirmed IBS record increased the specificity by 11 percentage points but has little meaning for clinical practice because it is an artificial patient population. Extending the follow-up time to allow for late recordings of IBD had no impact on sensitivity and specificity. The ROC analysis revealed that the recommended FC threshold of 50µg/g prioritises sensitivity over specificity.

Changing the threshold from 50µg/g to 100µg/g had a greater effect on the specificity than the sensitivity. For clinical practice this could translate into fewer referrals of non-IBD patients to gastroenterology at the expense of missing an additional nearly 7% of IBD cases. This interpretation is purely based on the logic of test results. Investigation of the proportion of referrals of patients with true positive, false positive, false negative and true negative test results showed that the actual management of tested patients may not clearly follow the expected behaviour of referring positives and managing test negative patients.

This interpretation assumes that referrals were recorded consistently for those with and without an IBD record and irrespective of FC test outcome. We interpret the findings with caution because we could not confirm the final classification of more than half of the tested patients and missing IBD cases cannot be excluded. Furthermore, all IBD patients would have been eventually referred according to the definition of an IBD diagnosis, which assumed that IBD diagnoses are only recorded following confirmation by a gastroenterologist and invasive testing. 61/210 (29.0%) IBD cases were either referred after 6 weeks or their referral was not recorded.

Strengths and limitations

One benefit of using routine data to estimate a test's accuracy is that the sample size is likely to be larger than the usual cross-sectional studies used to evaluate a test. This is particularly useful for target disorders such as IBD which have a low prevalence.[25] We applied strict eligibility criteria to ensure the reliability of reported test accuracy measures and we explored the effects of different follow-up times on the definition of IBD disease. In the case of FC testing in routine primary care, where it is not feasible to expose all tested patients to colonoscopy for disease verification, we were able to provide an answer to the question of the accuracy of the FC test as it is used in UK general practice. Our study shows

For peer review only - http://bmjopen.bmj.com/site/about/guidelines.xhtml

that FC testing together with the general practitioner's decision-making is an effective tool to exclude IBD in test negative patients.

The study may have suffered from selection bias because the inclusion criterion was a recorded FC test rather than symptomatic patients.[26] However, we believe this to be minimal when considering the point estimates and confidence intervals of test accuracy measures in the sensitivity analysis of patients with eligible symptoms.

The exclusion of close to half of the FC tests without a numerical test result raises concerns over the generalisability of the findings because excluded tests were statistically different to included tests in some variables tested. However, the tests were highly powered because of the large dataset and the clinical significance of the differences is highly uncertain.

Although imputation methods are often used to mitigate against missing data these were not implemented for three reasons. Firstly, in observational data, observed variables are not sufficient to account for the differences between missing and observed values.[27] Secondly, for imputation methods to be meaningful the proportion of missing data should be small and the missing subpopulation should be sufficiently similar to the wider population neither of which could be confirmed. Thirdly, in order to build reliable imputation models, more meaningful variables are needed including severity of symptoms, assay type, referral to gastroenterology and information on disease verification method, none of which is available in the IMRD-UK database. Therefore, potential biases could only be addressed by sensitivity analyses.[27]

The proportion of patients without a diagnosis following testing was large and could be due to any of the following: missed IBD; unrecorded IBS; other conditions not considered as study variables; or diagnoses recorded as free text and thus not available to the study. Also the research literature was too heterogeneous to be able to estimate the prevalence of other conditions accurately. For example, across 5 studies[28-32] the number of conditions varied widely and the prevalence ranged from 4% (16/399)[28] to 61% (422/694).[31] We were unable to predict IBD using other variables, such as symptoms and co-morbidities, in the non-IBD group due to the lack of strong predictors of IBD among the study variables. Without any further information on the characteristics of the non-IBD cases their true disease status remains uncertain.

Colonoscopy with histology is the preferred reference standard for diagnosis of IBD. However, information on secondary care events is not fully captured in the IMRD. In the

For peer review only - http://bmjopen.bmj.com/site/about/guidelines.xhtml

absence of sufficient information on colonoscopies, we used a combination of Read and drug codes as a reference standard under the assumption that IBD is not generally coded without confirmatory colonoscopy. Our pragmatic study for test accuracy aimed to establish the test performance of FC testing as it is used in clinical practice. The ideal reference standard is generally not feasible in these studies. While this does not invalidate the study, the study aim and the performance of the reference standard needs to be considered in the interpretation of the study findings.

We were interested in the broad category of inflammatory bowel disease. Whilst ulcerative colitis and Crohn's disease are the two predominant diagnoses included as part of IBD they are considered as the two extremes of a spectrum of chronic gut disorders.[33-35] IBD cannot be classified in 20% to 30% of patients at first presentation resulting in IBD unclassified cases.[33, 34] We also included unclassified forms of IBD such as indeterminate colitis and microscopic colitis to present the complete picture of IBD in primary care.

Our results are broadly in line with pooled estimates from a meta-analysis of 14 secondary and primary are studies,[11] which may suggest that the pooled test accuracy measures are applicable to the FC tested population in primary care. At the thresholds investigated, our sensitivity and specificity estimates are within the range of estimates from published primary care studies[28, 36, 37] However, previous primary care studies included small numbers of cases while our study provides narrower confidence intervals around test accuracy estimates. Crucially, our study shows that the specificity of 94% (95% CI 73% to 99%) reported in the NICE guidance is not applicable to the primary care setting,[8] where the broader definition of the non-IBD group means that the specificity and the positive predictive value of FC testing is much lower. Therefore, the interpretation of a test positive result may be challenging. Furthermore, the higher number of patients with a false positive result may not achieve the reduction of referrals to colonoscopy as anticipated by NICE guidance.

Implications for clinical practice

This study indicates that FC testing, when used by GPs, does not miss many cases of IBD in a low prevalence setting (NPV 99.6% [95% CI 99.3% to 99.7%]). GPs could, therefore, use the test confidently in ruling out IBD if the test is negative. However, the test does not perform as well in identifying IBD as modelled by national guidance. The positive predictive value of the FC test in identifying IBD is low, as essentially FC is a marker of intestinal inflammation rather than a specific marker for IBD.

For peer review only - http://bmjopen.bmj.com/site/about/guidelines.xhtml

The data also suggested that the higher threshold of 100µg/g may be appropriate for routine primary care. This is under the understanding that the test is not used in isolation and the objective is to reduce the number of unnecessary colonoscopies. Missed IBD patients would be assumed to re-present and be re-considered for referral within an appropriate time frame. However, the latest FC pathway recommends two cut-offs (100µg/g and 250µg/g)[10, 14] with an intermediate test range for retesting. The additional IBD cases missed at initial testing with this strategy may be inappropriately high. In this study we have not considered the feasibility of this latest pathway but it is likely to be more difficult to interpret for general practitioners.

Our study identified a number of inconsistencies between the intended use by national guidelines and the actual use by general practitioners. Firstly, general practitioners tested an unexpectedly high proportion of IBS patients which may suggest that general practitioners use the test to reassure patients with IBS. Secondly, practitioners referred proportionately more patients with false negative results than false positive and true negative results. This suggests that practitioners use other cues for referral rather than test results alone. Thirdly, close to half of all negative FC tested patients were referred, which suggests that general practitioners and/or patients may have limited confidence in the test. The current guidelines may not be applicable to general practice and the modelled cost-effectiveness of FC testing in general practice may be overoptimistic. When developing national guidance models should more closely represent general practice, rather than simply extrapolating from secondary care. This study shows for the first time the benefits and shortcomings of the performance of FC testing in a large dataset of primary care patients. It should help improve GPs' confidence in their diagnostic ability in this area and in test use and interpretation – especially where there is a negative test.

For peer review only

For peer review only - http://bmjopen.bmj.com/site/about/guidelines.xhtml

REFERENCES

1. Molodecky NA, Soon IS, Rabi DM, Ghali WA, Ferris M, Chernoff G, Benchimol EI, Panaccione R, Ghosh S, Barkema HW, Kaplan GG. Increasing incidence and prevalence of the inflammatory bowel diseases with time, based on systematic review. *Gastroenterology*. 2012;142(1):46-54. doi: 10.1053/j.gastro.2011.10.001.
2. King D, Reulen RC, Thomas T, Chandan JS, Thayakaran R, Subramanian A, Gokhale K, Bhala N, Nirantharakumar K, Adderley NJ, Trudgill N. Changing patterns in the epidemiology and outcomes of inflammatory bowel disease in the United Kingdom: 2000-2018. *Aliment Pharmacol Ther*. 2020;51(10):922-34. doi: 10.1111/apt.15701.
3. Schroder O, Naumann M, Shastri Y, Povse N, Stein J. Prospective evaluation of faecal neutrophil-derived proteins in identifying intestinal inflammation: combination of parameters does not improve diagnostic accuracy of calprotectin. *Aliment Pharmacol Ther*. 2007;26(7):1035-42. doi: 10.1111/j.1365-2036.2007.03457.x.
4. Lichtenstein GR, Loftus EV, Isaacs KL, Regueiro MD, Gerson LB, Sands BE. ACG clinical guideline: management of Crohn's disease in adults. *Am J Gastroenterol*. 2018;113:481. doi: 10.1038/ajg.2018.27 https://www.nature.com/articles/ajg201827#supplementary-information.
5. The Gastroenterological Society of Australia. Australian guidelines for general practitioners and physicians: inflammatory bowel disease. 2017 [cited 08/10/2018]; Available from: http://cart.gesa.org.au/membes/files/Clinical%20Guidelines%20and%20Updates/2017_IBD_guidelines_DRAFT_for_consultation_20102017.pdf.
6. Preiß J, Bokemeyer B, Buhr H, Dignaß A, Häuser W, Hartmann F, Herrlinger K, Kaltz B, Kienle P, Kruis W, Kucharzik T, Langhorst J, Schreiber S, Siegmund B, Stallmach A, Stange E, Stein J, Hoffmann J. Updated German clinical practice guideline on "Diagnosis and treatment of Crohn's disease". 2014 [cited 08/10/2018]; Available from: https://www.dgvs.de/wp-content/uploads/2016/11/DGVS_Empfehlung_fuer_Diagnistik_und_Therapie_des_Morbus_Crohn.pdf.
7. Maaser C, Sturm A, Vavricka SR, Kucharzik T, Fiorino G, Annese V, Calabrese E, Baumgart DC, Bettenworth D, Borralho Nunes P, Burisch J, Castiglione F, Eliakim R, Ellul P, González-Lama Y, Gordon H, Halligan S, Katsanos K, Kopylov U, Kotze PG, Krustinš E, Laghi A, Limdi JK, Rieder F, Rimola J, Taylor SA, Tolan D, van Rheenen P, Verstockt B, Stoker J. ECCO-ESGAR Guideline for diagnostic assessment in IBD part 1: initial diagnosis, monitoring of known IBD, detection of complications. *J Crohns Colitis*. 2019;13(2):144-64. doi: 10.1093/ecco-jcc/jjy113.
8. National Institute for Health and Care Excellence. Faecal calprotectin diagnostic tests for inflammatory diseases of the bowel: NICE diagnostics guidance [DG11]. 2013 [cited 17/12/2015]; Available from: https://www.nice.org.uk/guidance/dg11.
9. Ramraj R, Garcia A, Mosen D, Waiwaiole L, Smith N. Utility of fecal calprotectin in evaluation of chronic gastrointestinal symptoms in primary care. *Clin Pediatr (Phila)*. 2018;57(9):1058-63. doi: 10.1177/0009922817744607.
10. CSO Faecal Calprotectin Working Group. Faecal calprotectin in primary care as a decision diagnostic for inflammatory bowel disease and irritable bowel syndrome. 2018 [cited 09/07/2018]; Available from: https://www.pcc-cic.org.uk/article/updated-guidance-help-gps-easily-diagnose-serious-bowel-conditions.
11. Freeman K, Willis BH, Fraser H, Taylor-Phillips S, Clarke A. Faecal calprotectin to detect inflammatory bowel disease: a systematic review and exploratory meta-analysis of test accuracy. *BMJ Open*. 2019;9(3):e027428-e. doi: 10.1136/bmjopen-2018-027428.
12. Kontopantelis E, Stevens RJ, Helms PJ, Edwards D, Doran T, Ashcroft DM. Spatial distribution of clinical computer systems in primary care in England in 2016 and implications for primary care electronic medical record databases: a cross-sectional population study. *BMJ Open*. 2018;8(2):e020738. doi: 10.1136/bmjopen-2017-020738.

For peer review only - http://bmjopen.bmj.com/site/about/guidelines.xhtml

13. Blak BT, Thompson M, Dattani H, Bourke A. Generalisability of The Health Improvement Network (THIN) database: demographics, chronic disease prevalence and mortality rates. *Inform Prim Care*. 2011;19(4):251-5. doi: 10.14236/jhi.v19i4.820.
14. Turvill J, Turnock D. Audit of the impact of the York faecal calprotectin care pathway on colonoscopy activity. *Frontline Gastroenterol*. 2019;0:1-5. doi: 10.1136/flgastro-2019-101315.
15. The University of Manchester. Clinical Codes Repository. [cited 16/04/2020]; Available from: https://clinicalcodes.rss.mhs.man.ac.uk/.
16. Harkness EF, Grant L, O'Brien SJ, Chew-Graham CA, Thompson DG. Using read codes to identify patients with irritable bowel syndrome in general practice: a database study. *BMC Fam Pract*. 2013;14:183-. doi: 10.1186/1471-2296-14-183.
17. Lewis JD, Brensinger C, Bilker WB, Strom BL. Validity and completeness of the General Practice Research Database for studies of inflammatory bowel disease. *Pharmacoepidemiol Drug Saf*. 2002;11(3):211-8. doi: 10.1002/pds.698.
18. R Core Team. R: A language and environment for statistical computing. R Foundation for Statistical Computing, Vienna, Austria. [computer program]. 2017. Available from: https://www.R-project.org/.
19. Wilson EB. Probable Inference, the Law of Succession, and Statistical Inference. *Journal of the American Statistical Association*. 1927;22(158):209-12. doi: 10.2307/2276774.
20. Koopman PAR. Confidence Intervals for the Ratio of Two Binomial Proportions. *Biometrics*. 1984;40(2):513-7. doi: 10.2307/2531405.
21. Venkatraman ES. A permutation test to compare receiver operating characteristic curves. *Biometrics*. 2000;56(4):1134-8. doi: 10.1111/j.0006-341x.2000.01134.x.
22. Pavlidis P, Chedgy FJ, Tibble JA. Diagnostic accuracy and clinical application of faecal calprotectin in adult patients presenting with gastrointestinal symptoms in primary care. *Scand J Gastroenterol*. 2013;48(9):1048-54. doi: http://dx.doi.org/10.3109/00365521.2013.816771.
23. Turvill J, Turnock D, Holmes H, Jones A, McLaughlan E, Hilton V, Marriott S. Evaluation of the clinical and cost-effectiveness of the York Faecal Calprotectin Care Pathway. *Frontline Gastroenterol*. 2018;9:285-94. doi: 10.1136/flgastro-2018-100962.
24. Walker G, Moore L, Heerasing N, Hendy P, Perry M, McDonald T, Debenham T, Bethune R, Bewshea C, Hyde C, Heap G, Singh A, Calvert C, Kennedy N, Goodhand J, Ahmad T. Faecal calprotectin effectively excludes inflammatory bowel disease in 789 symptomatic young adults with/without alarm symptoms: a prospective UK primary care cohort study. *Aliment Pharmacol Ther*. 2018;47. doi: 10.1111/apt.14563.
25. Holtman GA, Berger MY, Burger H, Deeks JJ, Donner-Banzhoff N, Fanshawe TR, Koshiaris C, Leeflang MM, Oke JL, Perera R, Reitsma JB, Van den Bruel A. Development of practical recommendations for diagnostic accuracy studies in low-prevalence situations. *J Clin Epidemiol*. 2019;114:38-48. doi: https://doi.org/10.1016/j.jclinepi.2019.05.018.
26. Oostenbrink R, Moons KGM, Bleeker SE, Moll HA, Grobbee DE. Diagnostic research on routine care data: prospects and problems. *J Clin Epidemiol*. 2003;56(6):501-6. doi: https://doi.org/10.1016/S0895-4356(03)00080-5.
27. Sterne JAC, White IR, Carlin JB, Spratt M, Royston P, Kenward MG, Wood AM, Carpenter JR. Multiple imputation for missing data in epidemiological and clinical research: potential and pitfalls. *BMJ*. 2009;338:b2393. doi: 10.1136/bmj.b2393.
28. Conroy S, Hale MF, Cross SS, Swallow K, Sidhu RH, Sargur R, Lobo AJ. Unrestricted faecal calprotectin testing performs poorly in the diagnosis of inflammatory bowel disease in patients in primary care. *J Clin Pathol*. 2018;71:316-22. doi: http://dx.doi.org/10.1136/jclinpath-2017-204506.
29. Labaere D, Smismans A, Van Olmen A, Christiaens P, D'Haens G, Moons V, Cuyle PJ, Frans J, Bossuyt P. Comparison of six different calprotectin assays for the assessment of inflammatory bowel disease. *United European Gastroenterol J*. 2014;2(1):30-7. doi: https://dx.doi.org/10.1177/2050640613518201.
30. De Sloovere MM, De Smet D, Baert FJ, Debrabandere J, Vanpoucke HJ. Analytical and diagnostic performance of two automated fecal calprotectin immunoassays for detection

For peer review only - http://bmjopen.bmj.com/site/about/guidelines.xhtml

of inflammatory bowel disease. *Clin Chem Lab Med*. 2017;55(9):1435-46. doi: https://dx.doi.org/10.1515/cclm-2016-0796.
31. Mowat C, Digby J, Strachan JA, Wilson R, Carey FA, Fraser CG, Steele RJ. Faecal haemoglobin and faecal calprotectin as indicators of bowel disease in patients presenting to primary care with bowel symptoms. *Gut*. 2016;65(9):1463-9. doi: https://dx.doi.org/10.1136/gutjnl-2015-309579.
32. Oyaert M, Trouve C, Baert F, De Smet D, Langlois M, Vanpoucke H. Comparison of two immunoassays for measurement of faecal calprotectin in detection of inflammatory bowel disease: (pre)-analytical and diagnostic performance characteristics. *Clin Chem Lab Med*. 2014;52(3):391-7. doi: https://dx.doi.org/10.1515/cclm-2013-0699.
33. Geboes K, De Hertogh G. Indeterminate colitis. *Inflamm Bowel Dis*. 2003;9(5):324-31. doi: 10.1097/00054725-200309000-00007.
34. Odze RD. A contemporary and critical appraisal of 'indeterminate colitis'. *Mod Pathol*. 2015;28 Suppl 1:S30-46. doi: 10.1038/modpathol.2014.131.
35. Shrestha S, Olén O, Eriksson C, Everhov ÅH, Myrelid P, Visuri I, Ludvigsson JF, Schoultz I, Montgomery S, Sachs MC, Halfvarson J, Olsson M, Hjortswang H, Bengtsson J, Strid H, Andersson M, Jäghult S, Eberhardson M, Nordenvall C, Björk J, Fagerberg UL, Rejler M, Grip O, Karling P, Block M, Angenete E, Hellström PM, Gustavsson A. The use of ICD codes to identify IBD subtypes and phenotypes of the Montreal classification in the Swedish National Patient Register. *Scandinavian Journal of Gastroenterology*. 2020;55(4):430-5. doi: 10.1080/00365521.2020.1740778.
36. Boyd A, Peplow C, Dhaliwal A, O'Rourke J, Davies J, Milestone A. Primary care faecal calprotectin pathway: a three year analysis. *Colorectal Disease*. 2016;18:27. doi: http://dx.doi.org/10.1111/codi.13444.
37. Hogberg C, Karling P, Rutegard J, Lilja M. Diagnosing colorectal cancer and inflammatory bowel disease in primary care: the usefulness of tests for faecal haemoglobin, faecal calprotectin, anaemia and iron deficiency. A prospective study. *Scand J Gastroenterol*. 2017;52(1):69-75. doi: https://dx.doi.org/10.1080/00365521.2016.1228120.

For peer review only

For peer review only - http://bmjopen.bmj.com/site/about/guidelines.xhtml

FIGURE LEGEND

Figure 1 Flow diagram of inclusion criteria of FC tested patients into test accuracy study

Figure 2 ROC curve of sensitivity and false positive rate (1-specificity) for thresholds 33-300μg/g for the clinical question IBD versus non-IBD for tests with at least 6 months (N=5,970), 12 months (N=4,793) and 24 months (N=2,662) follow-up available after the FC test date

Figure 3 Sensitivity and specificity at thresholds 33-300μg/g for IBD versus non-IBD and IBD recorded within 6 months following the FC test date (N=5,970)

For peer review only

For peer review only - http://bmjopen.bmj.com/site/about/guidelines.xhtml

Author contributions

KF, STP, BHW, RR and AC designed the study. RR extracted the data from IMRD-UK and created the datasets. KF, STP and BHW carried out the analysis. KF, BHW, STP and AC contributed to the interpretation of the findings. KF drafted the manuscript. All authors critically revised the manuscript and approved the final version. KF takes responsibility for the integrity and accuracy of the data analysis. KF acts as guarantor. The corresponding author attests that all listed authors meet authorship criteria and that no others meeting the criteria have been omitted.

Acknowledgement

We would like to thank the patient advisory group of the project 'What is the role of faecal calprotectin testing in primary care" for their input.

Funding

KF is funded by a National Institute for Health Research (NIHR) DRF award (DRF-2016-09-038) for this research project. AC is supported by the National Institute for Health Research (NIHR) Applied Research Collaboration (ARC) West Midlands. This report presents independent research funded by the National Institute for Health Research (NIHR). The views expressed are those of the author and not necessarily those of the NHS, the NIHR or the Department of Health and Social Care. The funder had no role in the study design, data collection, data analysis and interpretation, writing of the report or the decision to submit for publication.

Competing interests

All authors have completed the Unified Competing Interest form (available on request from the corresponding author) and declare: KF is funded by the NIHR through a doctoral research fellowship. STP reports grants from the NIHR fellowship for Karoline Freemen. AC is supported by the NIHR ARC West Midlands initiative. BHW received grants from the Medical Research Council. RR has no conflicts to declare.

Data sharing statement

The IMRD-UK dataset cannot be shared under the data sharing agreement with the University of Birmingham on behalf of IQVIA.

Transparency

The lead author and manuscript's guarantor (KF) affirms that the manuscript is an honest, accurate and transparent account of the study being reported; that no important aspects of

For peer review only - http://bmjopen.bmj.com/site/about/guidelines.xhtml

the study have been omitted; and that any discrepancies from the study as planned have been explained.

Ethical approval

The study was approved by the Independent Scientific Advisory Committee (protocol number 17THIN089).

Dissemination declaration

We will disseminate the results to national charities of IBD and IBS patients.

Exclusive licence

The Corresponding Author has the right to grant on behalf of all authors and does grant on behalf of all authors, an exclusive licence (or non exclusive for government employees) on a worldwide basis to the BMJ Publishing Group Ltd to permit this article (if accepted) to be published in BMJ editions and any other BMJPGL products and sublicences such use and exploit all subsidiary rights, as set out in BMJ licence.

For peer review only

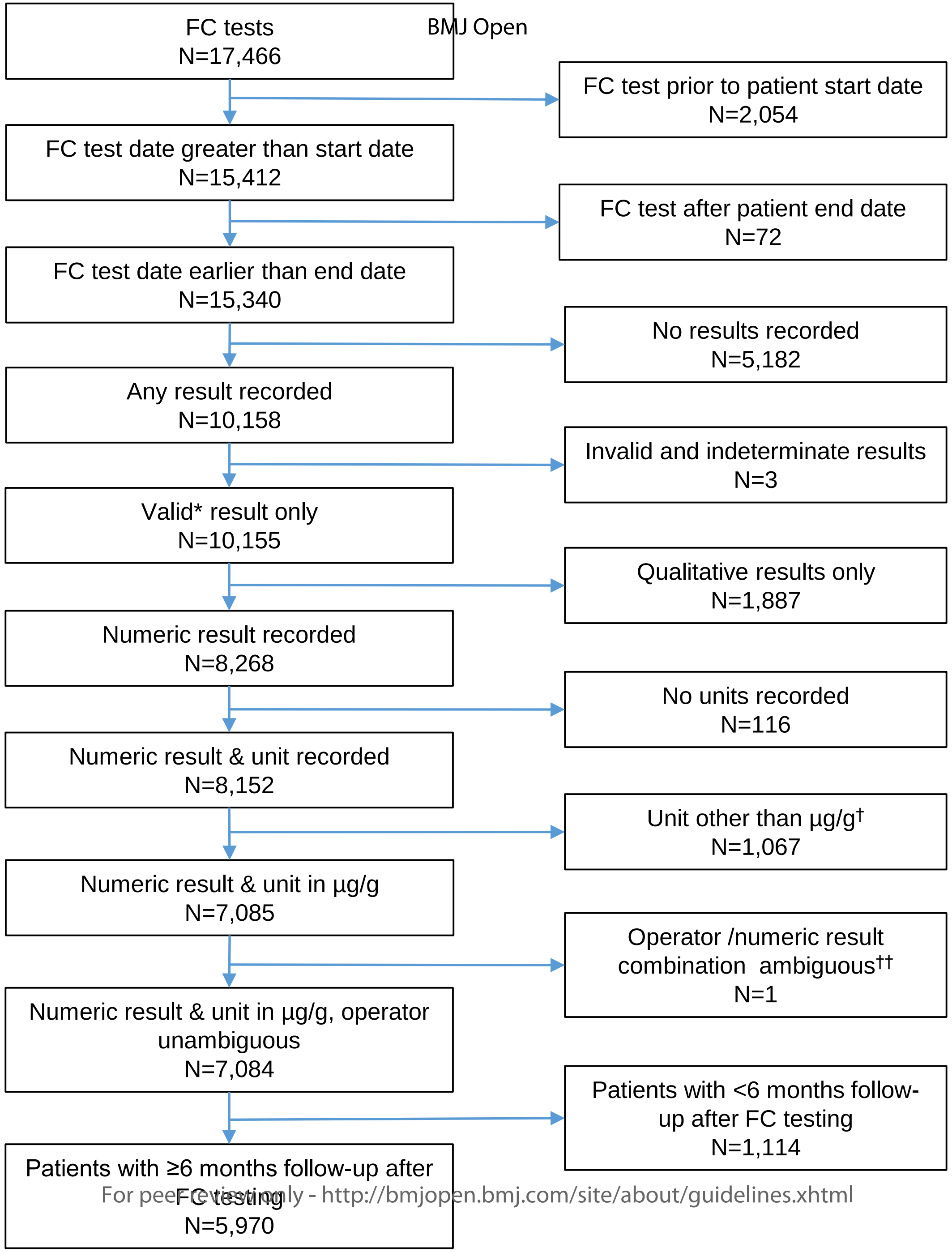

For peer review only - http://bmjopen.bmj.com/site/about/guidelines.xhtml

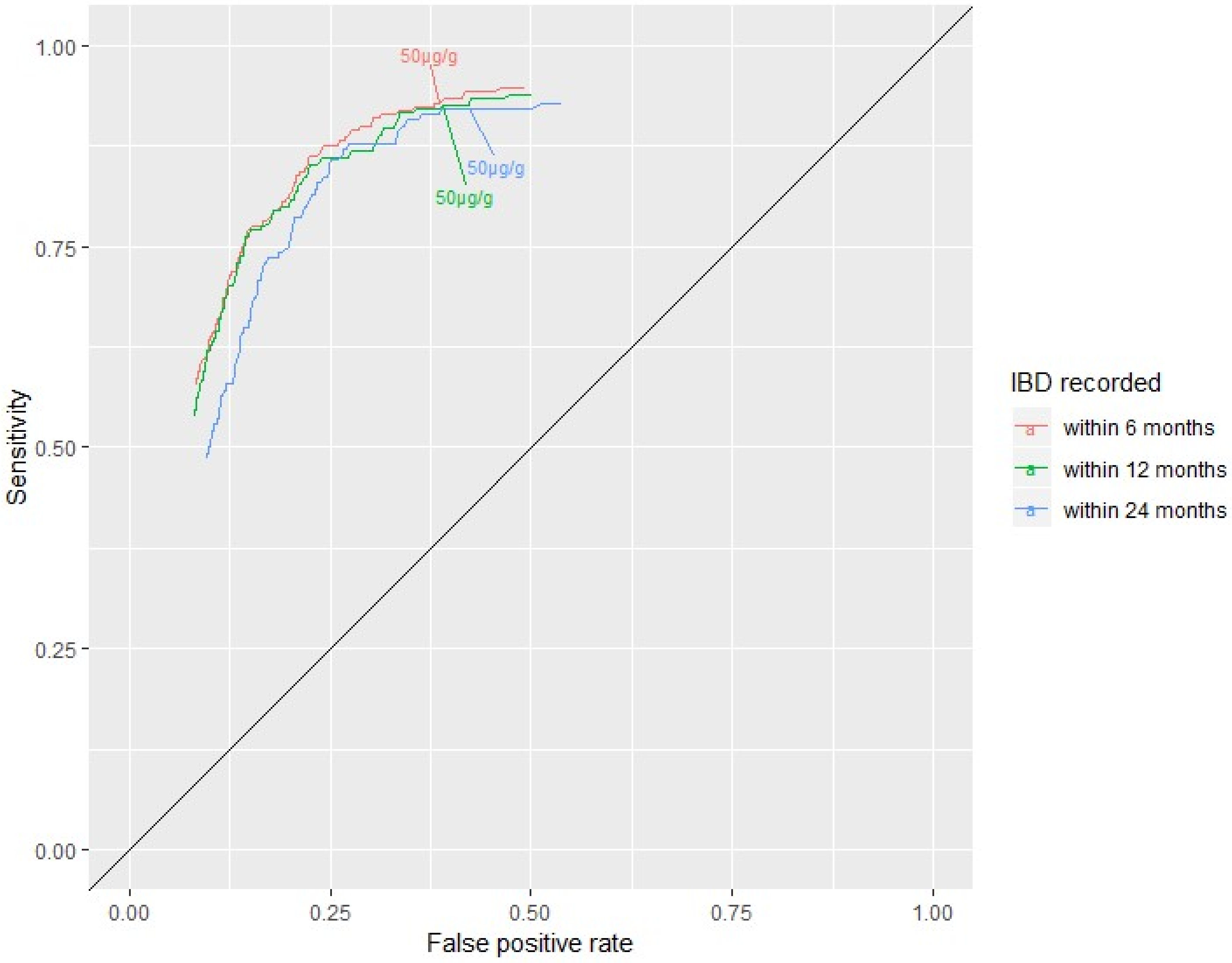

50µg/g
50µg/g
50µg/g
1.00
0.75
0.50
0.25
0.00
Sensitivity
0.00
0.25
0.50
0.75
1.00
False positive rate
IBD recorded
within 6 months
within 12 months
within 24 months

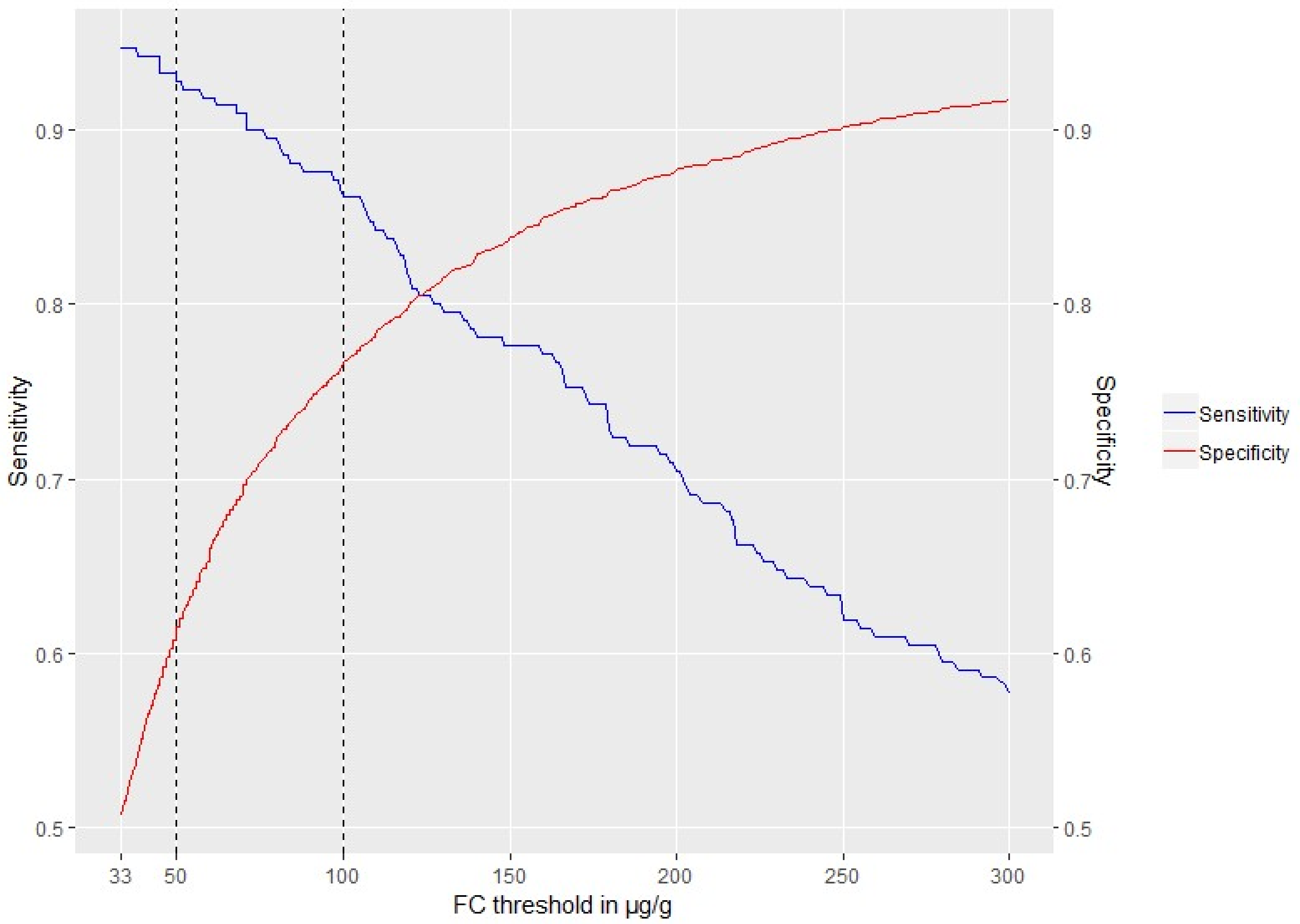
0.9
0.8
0.7
0.6
0.5
Sensitivity
Specificity
33
50
100
150
200
250
300
FC threshold in µg/g
Sensitivity
Specificity

SUPPLEMENT

Supplementary Table 1 Final variable code lists

Variable	medcode	description
IBD	14C4.11	H/O: ulcerative colitis
	J08z900	Orofacial Crohn's disease
	J4...12	Inflammatory bowel disease
	J40..00	Regional enteritis - Crohn's disease
	J40..11	Crohn's disease
	J40..12	Granulomatous enteritis
	J400.00	Regional enteritis of the small bowel
	J400000	Regional enteritis of the duodenum
	J400100	Regional enteritis of the jejunum
	J400200	Crohn's disease of the terminal ileum
	J400300	Crohn's disease of the ileum unspecified
	J400400	Crohn's disease of the ileum NOS
	J400500	Exacerbation of Crohn's disease of small intestine
	J400z00	Crohn's disease of the small bowel NOS
	J401.00	Regional enteritis of the large bowel
	J401000	Regional enteritis of the colon
	J401100	Regional enteritis of the rectum
	J401200	Exacerbation of Crohn's disease of large intestine
	J401z00	Crohn's disease of the large bowel NOS
	J401z11	Crohn's colitis
	J402.00	Regional ileocolitis
	J40z.11	Crohn's disease NOS
	J41..12	Ulcerative colitis and/or proctitis
	J410.00	Ulcerative proctocolitis
	J410000	Ulcerative ileocolitis
	J410100	Ulcerative colitis
	J410200	Ulcerative rectosigmoiditis
	J410300	Ulcerative proctitis
	J410400	Exacerbation of ulcerative colitis
	J410z00	Ulcerative proctocolitis NOS
	J411.00	Ulcerative (chronic) enterocolitis
	J412.00	Ulcerative (chronic) ileocolitis
	J413.00	Ulcerative pancolitis
	J436.00	Microscopic colitis
	J436000	Collagenous colitis
	J436100	Lymphocytic colitis
	J438.00	Left sided colitis
	J4z3.00	Non-infective colitis NOS
	J4z4.00	Non-infective sigmoiditis NOS
	J4z6.00	Indeterminate colitis

For peer review only

	Jyu4000	[X]Other Crohn's disease
	Jyu4100	[X]Other ulcerative colitis
	N031000	Arthropathy in ulcerative colitis
	N031100	Arthropathy in Crohn's disease
	N045300	Juvenile arthritis in Crohn's disease
	N045400	Juvenile arthritis in ulcerative colitis
	ZR3S.00	Crohn's disease activity index
	ZR3S.11	CDAI - Crohn's disease activity index
IBS	14CF.00	History of irritable bowel syndrome
	8CA4a00	Education about FODMAP exclusion diet
	8CA4Z00	Dietary education for irritable bowel syndrome
	8Cm..00	Management of irritable bowel syndrome
	Eu45324	[X]Psychogenic IBS
	J521.00	Irritable colon - Irritable bowel syndrome
	J521.11	Irritable bowel syndrome
	J521.13	Spastic colon
	J521000	Irritable bowel syndrome with diarrhoea
	J521100	Irritable bowel syndrome characterised by constipation
	J521200	IBS characterised by alternating bowel habit
Colorectal cancer	68W2400	Bowel scope (flexible sigmoidoscopy) screen: cancer detected
	68W2500	Bowel scope (flexi-sig) screen: suspected cancer detected
	9Ow1.00	Bowel cancer detected by national screening programme
	B13..00	Malignant neoplasm of colon
	B130.00	Malignant neoplasm of hepatic flexure of colon
	B131.00	Malignant neoplasm of transverse colon
	B132.00	Malignant neoplasm of descending colon
	B133.00	Malignant neoplasm of sigmoid colon
	B134.00	Malignant neoplasm of caecum
	B134.11	Carcinoma of caecum
	B136.00	Malignant neoplasm of ascending colon
	B137.00	Malignant neoplasm of splenic flexure of colon
	B138.00	Malignant neoplasm, overlapping lesion of colon
	B139.00	Hereditary nonpolyposis colon cancer
	B13y.00	Malignant neoplasm of other specified sites of colon
	B13z.00	Malignant neoplasm of colon NOS
	B13z.11	Colonic cancer
	B14..00	Malignant neoplasm of rectum, rectosigmoid junction and anus
	B140.00	Malignant neoplasm of rectosigmoid junction
	B141.00	Malignant neoplasm of rectum
	B141.11	Carcinoma of rectum
	B141.12	Rectal carcinoma
	B142000	Malignant neoplasm of cloacogenic zone

For peer review only

	B14y.00	Malig neop other site rectum, rectosigmoid junction and anus
	B14z.00	Malignant neoplasm rectum,rectosigmoid junction and anus NOS
	B1z0.11	Cancer of bowel
	B575.00	Secondary malignant neoplasm of large intestine and rectum
	B575000	Secondary malignant neoplasm of colon
	B575100	Secondary malignant neoplasm of rectum
	B575z00	Secondary malig neop of large intestine or rectum NOS
	B902400	Neoplasm of uncertain behaviour of colon
	B902500	Neoplasm of uncertain behaviour of rectum
	BB5N.00	[M]Adenomatous and adenocarcinomatous polyps of colon
	BB5N.11	[M]Adenoma or or adenocarcinoma in polyposis coli
	BB5N100	[M]Adenocarcinoma in adenomatous polposis coli
	BB5Nz00	[M]Adenomatous or adenocarcinomatous polyps of the colon NOS
	ZV10017	[V]Personal history of malignant neoplasm of rectum
Change in bowel habit	19EA.00	Change in bowel habit
	19EA.11	Altered bowel habit
	19EE.00	Increased frequency of defaecation
	J521200	IBS characterised by alternating bowel habit
	R078.00	[D]Change in bowel habit
Constipation	19C..00	Constipation
	19C..11	Constipation symptom
	19C..12	Costive symptom
	19C2.00	Constipated
	19CZ.00	Constipation NOS
	E264500	Psychogenic constipation
	J520.00	Constipation - functional
	J520000	Acute constipation
	J520100	Chronic constipation with overflow
	J520200	Chronic constipation without overflow
	J520400	Chronic constipation
	J520y00	Other specified constipation
	J520z00	Constipation NOS
	J521100	Irritable bowel syndrome characterised by constipation
	J52y100	Difficulty in ability defaecat
Diarrhoea	19E3.00	Incontinent of faeces
	19E3.11	Incontinent of faeces symptom
	19EE.00	Increased frequency of defaecation
	19EF.00	Urgent desire for stool
	19F..00	Diarrhoea symptoms
	19F..11	Diarrhoea

For peer review only

	19F..12	Loose stools
	19F2.00	Diarrhoea
	19F3.00	Spurious (overflow) diarrhoea
	19F5.00	Time since last episode of diarrhoea
	19FZ.00	Diarrhoea symptom NOS
	19FZ.11	Diarrhoea & vomiting, symptom
	19G..00	Diarrhoea and vomiting
	E264300	Psychogenic diarrhoea
	E264311	Spurious diarrhoea
	Eu45317	[X]Psychogenic diarrhoea
	J4...13	Noninfective diarrhoea
	J43z.11	Chronic diarrhoea
	J4z..11	Presumed noninfectious diarrhoea
	J4zz.11	Diarrhoea - presumed non-infectious
	J521000	Irritable bowel syndrome with diarrhoea
	J525.00	Functional diarrhoea
	J528.00	Intestinal hurry
	R077100	[D] Stools loose
Bloating	19A..00	Abdominal distension symptom
	19A2.00	Abdomen feels bloated
	19A3.00	Abdomen feels distended
	19A4.00	Abdomen feels swollen
	19AZ.00	Abd. distension symptom NOS
	19B..00	Flatulence/wind
	19B..12	Bloating symptom
	19B..14	Flatulence symptom
	19B..15	Wind symptom
	19B2.00	Excessive flatulence
	19B5.00	Excessive flatus
	19BZ.00	Wind NOS
	Eu45320	[X]Psychogenic flatulence
	R073.00	[D]Flatulence, eructation and gas pain
	R073000	[D]Flatulence
	R073200	[D]Gas pain (abdominal)
	R073300	[D]Abdominal distension, gaseous
	R073400	[D]Bloating
	R073500	[D]Tympanites (abdominal)
	R073z00	[D]Flatulence, eructation and gas pain NOS
	R073z11	[D]Wind
Abdominal pain	196..11	Abdominal pain type
	1962.00	Colicky abdominal pain
	1963.00	Non-colicky abdominal pain

For peer review only

	1967.00	Abdominal migraine - symptom
	1968.00	Abdominal discomfort
	1969.00	Abdominal pain
	1969000	Abdominal wall pain
	197..11	Flank pain
	197..12	Iliac fossa pain
	197..13	Site of abdominal pain
	197..14	Subcostal pain
	1971.00	Central abdominal pain
	1972.00	Epigastric pain
	1973.00	Left subcostal pain
	1974.00	Right subcostal pain
	1975.00	Left flank pain
	1976.00	Right flank pain
	1977.00	Right iliac fossa pain
	1978.00	Left iliac fossa pain
	1979.00	Suprapubic pain
	197A.00	Generalised abdominal pain
	197A.11	General abdominal pain-symptom
	197B.00	Upper abdominal pain
	197C.00	Lower abdominal pain
	197D.00	Right upper quadrant pain
	25C..00	O/E - abdo. pain on palpation
	25C..11	O/E - epigastric pain on palp.
	25C..12	O/E - iliac pain on palpation
	25C..14	O/E - umbilical pain on palp.
	25C..15	O/E - abdomen tender
	25C2.00	O/E - abd.pain-R.hypochondrium
	25C3.00	O/E - abd. pain - epigastrium
	25C4.00	O/E - abd.pain-L.hypochondrium
	25C5.00	O/E - abd. pain - R.lumbar
	25C6.00	O/E - abd. pain - umbilical
	25C7.00	O/E - abd. pain - L.lumbar
	25C8.00	O/E - abd. pain - R.iliac
	25C9.00	O/E - abd. pain - hypogastrium
	25CA.00	O/E - abd. pain - L.iliac
	25CZ.00	O/E -abd.pain on palpation NOS
	F262200	Abdominal migraine
	R073200	[D]Gas pain (abdominal)
	R090.00	[D]Abdominal pain
	R090000	[D]Abdominal tenderness
	R090100	[D]Abdominal colic
	R090400	[D]Abdominal cramps
	R090500	[D]Epigastric pain

For peer review only

	R090600	[D]Umbilical pain
	R090700	[D]Hypochondrial pain
	R090800	[D]Suprapubic pain
	R090900	[D]Pain in right iliac fossa
	R090A00	[D]Pain in left iliac fossa
	R090C00	[D]Loin pain
	R090D00	[D]Abdominal migraine
	R090E00	[D]Recurrent acute abdominal pain
	R090F00	[D]Acute abdomen
	R090G11	[D] Pelvic pain
	R090H00	[D]Upper abdominal pain
	R090J00	[D]Right upper quadrant pain
	R090K00	[D]Left upper quadrant pain
	R090L00	[D]Left lower quadrant pain
	R090M00	[D]Right lower quadrant pain
	R090N00	[D]Nonspecific abdominal pain
	R090P00	[D]Functional abdominal pain syndrome
	R090y00	[D]Other specified abdominal pain
	R090z00	[D]Abdominal pain NOS
	Ryu1000	[X]Pain localized to other parts of lower abdomen
	Ryu1100	[X]Other and unspecified abdominal pain
FC test	47J..00	Faecal calprotectin content
	47J0.00	Faecal calprotectin test indeterminate
	47J1.00	Faecal calprotectin test invalid
	47J2.00	Faecal calprotectin test positive
	47J3.00	Faecal calprotectin test negative
	4Q3J.00	Calprotectin level
**Variable**	**Drug code**	**Description**
Mesalazine	53913979	Mesalazine 800mg gastro-resistant tablets
	88517998	Mesalazine 400mg gastro-resistant tablets
	93624997	Mesalazine 250mg modified release tablets
	99583998	Mesalazine 250mg modified-release tablet
	54552979	Mesalazine 400mg gastro-resistant tablets
	55164978	Mesalazine 4g modified-release granules sachets sugar free
	55165978	Mesalazine 4g modified-release granules sachets sugar free
	58800979	Mesalazine 1.2g gastro-resistant modified-release tablets
	60584979	Mesalazine 3g gastro-resistant modified-release granules sachets sugar free
	60585979	Mesalazine 3g gastro-resistant modified-release granules sachets sugar free
	64868979	Mesalazine 2g modified-release granules sachets sugar free
	76424978	Mesalazine 1g modified-release tablets
	79867978	Mesalazine 400mg gastro-resistant tablets
	80928998	Mesalazine 3g gastro-resistant modified-release granules sachets sugar free

	80929998	Mesalazine 3g gastro-resistant modified-release granules sachets sugar free
	81193998	Mesalazine 1g modified-release tablets
	81194998	Mesalazine 1g modified-release tablets
	81683998	Mesalazine 1g suppositories
	81689998	Mesalazine 500mg gastro-resistant tablets
	81690998	Mesalazine 500mg gastro-resistant tablets
	81772998	Mesalazine 800mg gastro-resistant tablets
	81868998	Mesalazine 400mg gastro-resistant tablets
	83503998	Mesalazine 1.5g gastro-resistant modified-release granules sachets sugar free
	83504998	Mesalazine 1.5g gastro-resistant modified-release granules sachets sugar free
	83743998	Mesalazine 2g modified-release granules sachets sugar free
	83987998	Mesalazine 2g modified-release granules sachets sugar free
	84059998	Mesalazine 1g gastro-resistant modified-release granules sachets sugar free
	84209998	Mesalazine 800mg gastro-resistant tablets
	84290998	Mesalazine 1.2g gastro-resistant modified-release tablets
	84291998	Mesalazine 1.2g gastro-resistant modified-release tablets
	85560998	Mesalazine 800mg gastro-resistant tablets
	87761998	Mesalazine 400mg gastro-resistant tablets
	87909998	Mesalazine 1g gastro-resistant modified-release granules sachets sugar free
	87910998	Mesalazine 500mg gastro-resistant modified-release granules sachets sugar free
	87911998	Mesalazine 500mg gastro-resistant modified-release granules sachets sugar free
	89992997	Mesalazine 2g/59ml enema
	89992998	Mesalazine 500mg suppositories
	92346998	Mesalazine 1g/application foam enema
	92347998	Mesalazine 400mg gastro-resistant tablets
	92764997	Mesalazine 1g modified-release granules sachets sugar free
	92764998	Mesalazine 500mg modified-release tablets
	93623996	Mesalazine 250mg suppositories
	93623997	Mesalazine 1g/application foam enema
	93623998	Mesalazine 1g/100ml enema
	93624996	Mesalazine 1g suppositories
	93624998	Mesalazine 1g/100ml enema
	93728992	Mesalazine 500mg modified-release tablets
	94564992	Mesalazine 500mg modified-release tablets
	95041990	Mesalazine 400mg gastro-resistant tablets
	95888997	Mesalazine 250mg gastro-resistant tablets
	95888998	Mesalazine 400mg gastro-resistant tablets
	96608996	Mesalazine 2g/59ml enema
	96608997	Mesalazine 1g suppositories
	96608998	Mesalazine 500mg suppositories
	96659996	Mesalazine 1g/application foam enema
	96659997	Mesalazine 500mg suppositories

For peer review only

	96659998	Mesalazine 250mg suppositories
	96883990	Mesalazine 400mg gastro-resistant tablets
	96916992	Mesalazine 500mg modified-release tablets
	97381998	Mesalazine 400mg gastro-resistant tablets
	97764998	Mesalazine 250mg gastro-resistant tablets
	98001992	Mesalazine 250mg gastro-resistant tablets
	99486979	Mesalazine 400mg gastro-resistant tablets
	99487979	Mesalazine 400mg gastro-resistant tablets
	99488979	Mesalazine 400mg gastro-resistant tablets
	99490979	Mesalazine 1g modified-release granules sachets sugar free
	99492979	Mesalazine 1g suppositories
	99494979	Mesalazine 1g suppositories
	99495979	Mesalazine 500mg modified-release tablets
	99498979	Mesalazine 500mg modified-release tablets
	99583996	Mesalazine 1g gastro-resistant modified-release granules sachets sugar free
	99583997	Mesalazine 500mg modified-release tablets
Olsalazine	92400998	Olsalazine 500mg tablets
	92401998	Olsalazine 250mg capsules
	94437997	Olsalazine 500mg tablets
	94437998	Olsalazine 250mg capsules
	94438997	Olsalazine 500mg tablets
	94438998	Olsalazine 250mg capsules
Balsalazide	88489998	Balsalazide 750mg capsules
	88492998	Balsalazide 750mg capsules
Sulfasalazine	89598997	Sulfasalazine 3g/100ml retention enema
	89598998	Sulfasalazine 500mg suppositories
	89604997	Sulfasalazine 3g/100ml retention enema
	89604998	Sulfasalazine 500mg suppositories
	95256996	Sulfasalazine 3g/100ml enema
	95256997	Sulfasalazine 500mg suppositories
	97281996	Sulfasalazine 3g/100ml retention enema
	97281997	Sulfasalazine 500mg suppositories
Beclometasone	86941998	Beclometasone 5mg gastro-resistant modified-release tablets
	86942998	Beclometasone 5mg gastro-resistant modified-release tablets
Budenoside	50988978	Budesonide 9mg modified-release tablets
	50989978	Budesonide 9mg modified-release tablets
	60586979	Budesonide 9mg gastro-resistant granules sachets
	60587979	Budesonide 9mg gastro-resistant granules sachets
	80931998	Budesonide 9mg gastro-resistant granules sachets
	80932998	Budesonide 9mg gastro-resistant granules sachets
	84636998	Budesonide 2mg foam enema
	84637998	Budesonide 2mg foam enema
	89238998	Budesonide 2mg/100ml enema
	89239998	Budesonide 2mg/100ml enema

For peer review only

	94125992	Cortenema enema ml liq
Prednisolone	60097979	Prednisolone 20mg/application foam enema
	84741998	Prednisolone 40mg/100ml enema
	90310979	Prednisolone 20mg/application foam enema
	93706998	Prednisolone sodium phosphate 5mg suppositories
	94336997	Prednisolone sodium phosphate 5mg suppositories
	94336998	Prednisolone rectal ointment
	94451998	Prednisolone 20mg/application foam enema
	94452998	Prednisolone 20mg/application foam enema
	94468998	Prednisolone 20mg/100ml enema standard tube
	98370998	Prednisolone sodium phosphate 5mg suppositories
	98371998	Prednisolone 20mg/100ml enema standard tube
	99227997	Prednisolone 20mg/100ml enema standard tube
	99227998	Prednisolone 20mg/100ml enema standard tube
	99388979	Prednisolone 20mg/100ml enema standard tube
	99389979	Prednisolone 20mg/100ml enema standard tube
	89284997	Cinchocaine 1mg / Prednisolone hexanoate 1.3mg suppositories

For peer review only

Supplementary Table 2 Patient characteristics with FC tests included and excluded from the test accuracy study

	Included FC tests (N=5970)	Excluded FC tests (N=9370)	p-value
Age (mean (SD)) at FC test date, range	44.07 (16.23), 18-101	43.42 (16.54), 18-97	0.01594
Sex (female (%))	3828 (64.1)	5963 (63.6)	0.5452
BMI (mean (SD))	26.86 (6.25)	26.93 (6.22)	0.5285
Not reported n (%)	656 (10.99)	1061 (11.32)	0.521
Ethnicity n (%)			
White	3441 (57.64)	5507 (58.77)	0.1647
Asian	93 (1.56)	249 (2.66)	<0.0001
Black	63 (1.06)	135 (1.44)	0.0392
Mixed	24 (0.40)	64 (0.68)	0.0246
Other	33 (0.55)	55 (0.59)	0.7844
Not reported	2316 (38.79)	3360 (35.86)	0.0002
Townsend score n (%)			
1 (least deprived)	988 (16.55)	1936 (20.66)	<0.0001
2	1101 (18.44)	1691 (18.05)	0.5362
3	1140 (19.10)	1720 (18.36)	0.2518
4	1135 (19.01)	1666 (17.78)	0.0542
5 (most deprived)	1062 (17.79)	1149 (12.26)	<0.0001
Missing	544 (9.11)	1208 (12.89)	<0.0001
Country (n) (%)			
England	3748 (62.78)	7073 (75.49)	<0.0001
Northern Ireland	16 (0.27)	566 (6.04)	<0.0001
Scotland	1916 (32.09)	1007 (10.75)	<0.0001
Wales	290 (4.86)	724 (7.73)	<0.0001
CRC ever recorded n (%)	31 (0.52)	45 (0.48)	0.7373
IBS ever recorded n (%)	1897 (31.78)	2649 (28.27)	<0.0001
IBD ever recorded n (%)	208 (3.48)	208 (2.22)	<0.0001
IBD + IBS recorded n (%)	78 (1.31)	63 (0.67)	<0.0001
IBD + CRC recorded n (%)	2 (0.03)	0 (0)	0.0764
No diagnosis recorded n (%)	3754 (62.88)	6405 (68.36)	<0.0001

There were regional differences, with more FC tests excluded from Wales and Northern Ireland and fewer excluded from Scotland and England. There were also more excluded from the least deprived group according to Townsend Score, and fewer excluded from the most deprived group.

Supplementary Table 3 Test accuracy measures of FC testing at threshold of 100µg/g compared to 50µg/g

Threshold	N	TP	FP	FN	TN	Sensitivity, % (95% CI)	Specificity, % (95% CI)	PPV, % (95% CI)	NPV, % (95% CI)
50µg/g	5970	195	2219	15	3541	92.9 (88.6 to 95.6)	61.5 (60.2 to 62.7)	8.1 (7.1 to 9.2)	99.6 (99.3 to 99.7)
100µg/g	5970	181	1344	29	4416	86.2 (80.9 to 90.2)	76.7 (75.6 to 77.7)	11.9 (10.3 to 13.6)	99.3 (99.1 to 99.5)
250µg/g	5970	130	563	80	5197	61.9 (55.2 to 68.2)	90.2 (89.4 to 91)	18.8 (16 to 21.8)	98.5 (98.1 to 98.8)

N sample size, TP true positives, FP false positives, FN false negatives, TN true negatives, PPV positive predictive value, NPV negative predictive value, CI confidence interval

For peer review only

Section & Topic	No	Item	Reported on page #
**TITLE OR ABSTRACT**			
	**1**	Identification as a study of diagnostic accuracy using at least one measure of accuracy (such as sensitivity, specificity, predictive values, or AUC)	1, 2
**ABSTRACT**			
	**2**	Structured summary of study design, methods, results, and conclusions (for specific guidance, see STARD for Abstracts)	2
**INTRODUCTION**			
	**3**	Scientific and clinical background, including the intended use and clinical role of the index test	4
	**4**	Study objectives and hypotheses	4
**METHODS**			
Study design	**5**	Whether data collection was planned before the index test and reference standard were performed (prospective study) or after (retrospective study)	5
Participants	**6**	Eligibility criteria	5
	**7**	On what basis potentially eligible participants were identified (such as symptoms, results from previous tests, inclusion in registry)	5
	**8**	Where and when potentially eligible participants were identified (setting, location and dates)	5
	**9**	Whether participants formed a consecutive, random or convenience series	NA all eligible patients in database were included
Test methods	**10a**	Index test, in sufficient detail to allow replication	5
	**10b**	Reference standard, in sufficient detail to allow replication	5 and Supplementary table 1
	**11**	Rationale for choosing the reference standard (if alternatives exist)	NA
	**12a**	Definition of and rationale for test positivity cut-offs or result categories of the index test, distinguishing pre-specified from exploratory	5
	**12b**	Definition of and rationale for test positivity cut-offs or result categories of the reference standard, distinguishing pre-specified from exploratory	5 and 6
	**13a**	Whether clinical information and reference standard results were available to the performers/readers of the index test	NA routine data
	**13b**	Whether clinical information and index test results were available to the assessors of the reference standard	NA routine data
Analysis	**14**	Methods for estimating or comparing measures of diagnostic accuracy	5 and 6
	**15**	How indeterminate index test or reference standard results were handled	Figure 1
	**16**	How missing data on the index test and reference standard were handled	5, Figure 1
	**17**	Any analyses of variability in diagnostic accuracy, distinguishing pre-specified from exploratory	6
	**18**	Intended sample size and how it was determined	NA all eligible patients from database included
**RESULTS**			
Participants	**19**	Flow of participants, using a diagram	Figure 1
	**20**	Baseline demographic and clinical characteristics of participants	Supplementary table 2
	**21a**	Distribution of severity of disease in those with the target condition	NA
	**21b**	Distribution of alternative diagnoses in those without the target condition	NA
	**22**	Time interval and any clinical interventions between index test and reference standard	NA
Test results	**23**	Cross tabulation of the index test results (or their distribution) by the results of the reference standard	Tables 1, 2, 3 and supplementary table 3
	**24**	Estimates of diagnostic accuracy and their precision (such as 95% confidence intervals)	Tables 1, 2, 3 and supplementary table 3
	**25**	Any adverse events from performing the index test or the reference standard	NA

For peer review only - http://bmjopen.bmj.com/site/about/guidelines.xhtml

cc BY SA

**DISCUSSION**			
	**26**	Study limitations, including sources of potential bias, statistical uncertainty, and generalisability	12-13
	**27**	Implications for practice, including the intended use and clinical role of the index test	14-15
**OTHER INFORMATION**			
	**28**	Registration number and name of registry	NA
	**29**	Where the full study protocol can be accessed	5
	**30**	Sources of funding and other support; role of funders	20

For peer review only

STARD 2015

AIM

STARD stands for "Standards for Reporting Diagnostic accuracy studies". This list of items was developed to contribute to the completeness and transparency of reporting of diagnostic accuracy studies. Authors can use the list to write informative study reports. Editors and peer-reviewers can use it to evaluate whether the information has been included in manuscripts submitted for publication.

EXPLANATION

A **diagnostic accuracy study** evaluates the ability of one or more medical tests to correctly classify study participants as having a **target condition.** This can be a disease, a disease stage, response or benefit from therapy, or an event or condition in the future. A medical test can be an imaging procedure, a laboratory test, elements from history and physical examination, a combination of these, or any other method for collecting information about the current health status of a patient.

The test whose accuracy is evaluated is called **index test.** A study can evaluate the accuracy of one or more index tests. Evaluating the ability of a medical test to correctly classify patients is typically done by comparing the distribution of the index test results with those of the **reference standard**. The reference standard is the best available method for establishing the presence or absence of the target condition. An accuracy study can rely on one or more reference standards.

If test results are categorized as either positive or negative, the cross tabulation of the index test results against those of the reference standard can be used to estimate the **sensitivity** of the index test (the proportion of participants *with* the target condition who have a positive index test), and its **specificity** (the proportion *without* the target condition who have a negative index test). From this cross tabulation (sometimes referred to as the contingency or "2x2" table), several other accuracy statistics can be estimated, such as the positive and negative **predictive values** of the test. Confidence intervals around estimates of accuracy can then be calculated to quantify the statistical **precision** of the measurements.

If the index test results can take more than two values, categorization of test results as positive or negative requires a **test positivity cut-off**. When multiple such cut-offs can be defined, authors can report a receiver operating characteristic (ROC) curve which graphically represents the combination of sensitivity and specificity for each possible test positivity cut-off. The **area under the ROC curve** informs in a single numerical value about the overall diagnostic accuracy of the index test.

The **intended use** of a medical test can be diagnosis, screening, staging, monitoring, surveillance, prediction or prognosis. The **clinical role** of a test explains its position relative to existing tests in the clinical pathway. A replacement test, for example, replaces an existing test. A triage test is used before an existing test; an add-on test is used after an existing test.

Besides diagnostic accuracy, several other outcomes and statistics may be relevant in the evaluation of medical tests. Medical tests can also be used to classify patients for purposes other than diagnosis, such as staging or prognosis. The STARD list was not explicitly developed for these other outcomes, statistics, and study types, although most STARD items would still apply.

DEVELOPMENT

This STARD list was released in 2015. The 30 items were identified by an international expert group of methodologists, researchers, and editors. The guiding principle in the development of STARD was to select items that, when reported, would help readers to judge the potential for bias in the study, to appraise the applicability of the study findings and the validity of conclusions and recommendations. The list represents an update of the first version, which was published in 2003.

More information can be found on http://www.equator-network.org/reporting-guidelines/stard.

For peer review only

CC BY SA

The RECORD statement – checklist of items, extended from the STROBE statement, that should be reported in observational studies using routinely collected health data.

	Item No.	STROBE items	Location in manuscript where items are reported	RECORD items	Location in manuscript where items are reported
**Title and abstract**					
	1	(a) Indicate the study's design with a commonly used term in the title or the abstract (b) Provide in the abstract an informative and balanced summary of what was done and what was found	p1-2	RECORD 1.1: The type of data used should be specified in the title or abstract. When possible, the name of the databases used should be included.<br><br>RECORD 1.2: If applicable, the geographic region and timeframe within which the study took place should be reported in the title or abstract.<br><br>RECORD 1.3: If linkage between databases was conducted for the study, this should be clearly stated in the title or abstract.	P1<br><br>p2<br><br>NA
**Introduction**					
Background rationale	2	Explain the scientific background and rationale for the investigation being reported	p4		
Objectives	3	State specific objectives, including any prespecified hypotheses	p4		
**Methods**					
Study Design	4	Present key elements of study design early in the paper	p1, 5		
Setting	5	Describe the setting, locations, and relevant dates, including periods of recruitment, exposure, follow-up, and data collection	P5		

For peer review only

Participants	6	*(a) Cohort study* - Give the eligibility criteria, and the sources and methods of selection of participants. Describe methods of follow-up<br>*Case-control study* - Give the eligibility criteria, and the sources and methods of case ascertainment and control selection. Give the rationale for the choice of cases and controls<br>*Cross-sectional study* - Give the eligibility criteria, and the sources and methods of selection of participants<br><br>*(b) Cohort study* - For matched studies, give matching criteria and number of exposed and unexposed<br>*Case-control study* - For matched studies, give matching criteria and the number of controls per case	p5-6<br><br>NA	RECORD 6.1: The methods of study population selection (such as codes or algorithms used to identify subjects) should be listed in detail. If this is not possible, an explanation should be provided.<br><br>RECORD 6.2: Any validation studies of the codes or algorithms used to select the population should be referenced. If validation was conducted for this study and not published elsewhere, detailed methods and results should be provided.<br><br>RECORD 6.3: If the study involved linkage of databases, consider use of a flow diagram or other graphical display to demonstrate the data linkage process, including the number of individuals with linked data at each stage.	Supplementary Table 1<br><br>p5<br><br>NA
Variables	7	Clearly define all outcomes, exposures, predictors, potential confounders, and effect modifiers. Give diagnostic criteria, if applicable.	p5-6	RECORD 7.1: A complete list of codes and algorithms used to classify exposures, outcomes, confounders, and effect modifiers should be provided. If these cannot be reported, an explanation should be provided.	Supplementary Table 1
Data sources/ measurement	8	For each variable of interest, give sources of data and details of methods of assessment (measurement).<br>Describe comparability of assessment methods if there is more than one group	p5		

For peer review only

Bias	9	Describe any efforts to address potential sources of bias	p6		
Study size	10	Explain how the study size was arrived at	NA		
Quantitative variables	11	Explain how quantitative variables were handled in the analyses. If applicable, describe which groupings were chosen, and why	p12		
Statistical methods	12	(a) Describe all statistical methods, including those used to control for confounding<br>(b) Describe any methods used to examine subgroups and interactions<br>(c) Explain how missing data were addressed<br>(d) *Cohort study* - If applicable, explain how loss to follow-up was addressed<br>*Case-control study* - If applicable, explain how matching of cases and controls was addressed<br>*Cross-sectional study* - If applicable, describe analytical methods taking account of sampling strategy<br>(e) Describe any sensitivity analyses	p6		
Data access and cleaning methods		..		RECORD 12.1: Authors should describe the extent to which the investigators had access to the database population used to create the study population.	p20

For peer review only

				RECORD 12.2: Authors should provide information on the data cleaning methods used in the study.	Figure 1
Linkage		..		RECORD 12.3: State whether the study included person-level, institutional-level, or other data linkage across two or more databases. The methods of linkage and methods of linkage quality evaluation should be provided.	NA
**Results**					
Participants	13	(a) Report the numbers of individuals at each stage of the study (*e.g.*, numbers potentially eligible, examined for eligibility, confirmed eligible, included in the study, completing follow-up, and analysed)<br>(b) Give reasons for non-participation at each stage.<br>(c) Consider use of a flow diagram	Figure 1	RECORD 13.1: Describe in detail the selection of the persons included in the study (*i.e.,* study population selection) including filtering based on data quality, data availability and linkage. The selection of included persons can be described in the text and/or by means of the study flow diagram.	Figure 1
Descriptive data	14	(a) Give characteristics of study participants (*e.g.*, demographic, clinical, social) and information on exposures and potential confounders<br>(b) Indicate the number of participants with missing data for each variable of interest<br>(c) *Cohort study* - summarise follow-up time (*e.g.*, average and total amount)	Supplementary Table 2		
Outcome data	15	*Cohort study* - Report numbers of outcome events or summary measures over time<br>*Case-control study* - Report numbers in each exposure	Tables 1 and 2		

For peer review only

		category, or summary measures of exposure<br>*Cross-sectional study* - Report numbers of outcome events or summary measures			
Main results	16	(a) Give unadjusted estimates and, if applicable, confounder-adjusted estimates and their precision (e.g., 95% confidence interval). Make clear which confounders were adjusted for and why they were included<br>(b) Report category boundaries when continuous variables were categorized<br>(c) If relevant, consider translating estimates of relative risk into absolute risk for a meaningful time period	p7-9		
Other analyses	17	Report other analyses done—e.g., analyses of subgroups and interactions, and sensitivity analyses	p9-10		
**Discussion**					
Key results	18	Summarise key results with reference to study objectives	p12		
Limitations	19	Discuss limitations of the study, taking into account sources of potential bias or imprecision. Discuss both direction and magnitude of any potential bias	p12-13	RECORD 19.1: Discuss the implications of using data that were not created or collected to answer the specific research question(s). Include discussion of misclassification bias, unmeasured confounding, missing data, and changing eligibility over time, as they pertain to the study being reported.	p12-13
Interpretation	20	Give a cautious overall interpretation of results considering objectives,	p14-15		

For peer review only

		limitations, multiplicity of analyses, results from similar studies, and other relevant evidence			
Generalisability	21	Discuss the generalisability (external validity) of the study results	p13-14		
**Other Information**					
Funding	22	Give the source of funding and the role of the funders for the present study and, if applicable, for the original study on which the present article is based	p20		
Accessibility of protocol, raw data, and programming code		..		RECORD 22.1: Authors should provide information on how to access any supplemental information such as the study protocol, raw data, or programming code.	p20

*Reference: Benchimol EI, Smeeth L, Guttmann A, Harron K, Moher D, Petersen I, Sørensen HT, von Elm E, Langan SM, the RECORD Working Committee. The REporting of studies Conducted using Observational Routinely-collected health Data (RECORD) Statement. *PLoS Medicine* 2015; in press.

*Checklist is protected under Creative Commons Attribution (CC BY) license.

For peer review only
